# The CUT&RUN suspect list of problematic regions of the genome

**DOI:** 10.1186/s13059-023-03027-3

**Published:** 2023-08-10

**Authors:** Anna Nordin, Gianluca Zambanini, Pierfrancesco Pagella, Claudio Cantù

**Affiliations:** 1 Wallenberg Centre for Molecular Medicine, Linköping University, Linköping, Sweden; 2 Department of Biomedical and Clinical Sciences, Division of Molecular Medicine and Virology, Faculty of Medicine and Health Sciences, Linköping University, Linköping, Sweden

**Keywords:** CUT&RUN, Chromatin, Bioinformatics, Peak calling, Blacklist, Suspect list

## Abstract

**Background:**

Cleavage Under Targets and Release Using Nuclease (CUT&RUN) is an increasingly popular technique to map genome-wide binding profiles of histone modifications, transcription factors, and co-factors. The ENCODE project and others have compiled blacklists for ChIP-seq which have been widely adopted: these lists contain regions of high and unstructured signal, regardless of cell type or protein target, indicating that these are false positives. While CUT&RUN obtains similar results to ChIP-seq, its biochemistry and subsequent data analyses are different. We found that this results in a CUT&RUN-specific set of undesired high-signal regions.

**Results:**

We compile suspect lists based on CUT&RUN data for the human and mouse genomes, identifying regions consistently called as peaks in negative controls. Using published CUT&RUN data from our and other labs, we show that the CUT&RUN suspect regions can persist even when peak calling is performed with SEACR or MACS2 against a negative control and after ENCODE blacklist removal. Moreover, we experimentally validate the CUT&RUN suspect lists by performing reiterative negative control experiments in which no specific protein is targeted, showing that they capture more than 80% of the peaks identified.

**Conclusions:**

We propose that removing these problematic regions can substantially improve peak calling in CUT&RUN experiments, resulting in more reliable datasets.

**Supplementary Information:**

The online version contains supplementary material available at 10.1186/s13059-023-03027-3.

## Background

Cleavage Under Targets and Release Using Nuclease (CUT&RUN, hereafter referred to as C&R) is a technique developed to map the genome-wide binding profiles of histone modifications, transcription factors and co-factors [1]. Like other genomics techniques, C&R uses short-read next generation sequencing (NGS) to generate datasets and thus depends on reliable mapping and genome assemblies for their accurate interpretation. Repetitive regions, assembly gaps, and other computational challenges can lead to the rise of sequencing artifacts, resulting in areas of significant signal enrichment that are not due to biological processes [2]. Additionally, high-signal regions can occur as consequence of several procedures, such as cell fixation or PCR-based amplification after adaptor ligation of sequencing libraries [3–5].

Sets of artifactual and high-signal regions that should be excluded from the analysis, known typically as blacklists, have previously been generated by the ENCODE consortium and others for chromatin immunoprecipitation followed by sequencing (ChIP-seq) [6, 7]. C&R and ChIP-seq obtain comparable results, and the ENCODE blacklist have been used for both these assays. However, C&R and ChIP rely on different biochemical procedures; for example, in ChIP-seq, the chromatin is crosslinked, sonicated, and immunoprecipitated, while in C&R, no crosslinking is applied and the target-associated DNA fragments are obtained by digestion with the MNase-proteinA (pA-MNase) or proteinA/G (pA/G-MNase) fusion proteins [1]. The determination of signal enrichment, peak calling, can also be done differently between the two techniques. While most ChIP-seq peak calling algorithms use local signal to background ratios to determine significance, the low background typical of C&R renders this difficult. This led to the development of the C&R specific peak caller SEACR, which uses global background (as opposed to local background) to determine a signal to noise threshold against which to call peaks [8]. Artifacts in ChIP-seq have been shown to bias peak calling results [2]; we reasoned that, due to its reliance on global background signal, SEACR may be even more sensitive to unspecific signals.

To address these differences between C&R and other NGS-based techniques, we compiled lists of suspect regions (suspect lists) for the hg38 human and mm10 mouse genomes built exclusively on C&R data. To determine regions that produce detectable signals in negative controls, hence likely to be erroneously called as peaks across experiments, we downloaded C&R negative control datasets (*N* = 20 per genome from 20 different cell types, from 40 different studies) and performed peak calling on them to identify peaks consistently identified by SEACR. This allowed us to establish a human and a mouse C&R suspect list of by-definition artifactual peaks. Both human and mouse C&R suspect lists obtained in this manner show high precision, as they encompass less than 0.2% of their respective genome yet succeed—when used to subtract the false peaks from C&R datasets—in increasing the genome-wide variance between samples, indicating that the suspect list regions are commonly enriched across C&R experiments, regardless of the target and the cell type. This indicates that these regions ought to be removed.

To validate the C&R suspect lists generated, we performed reiterative negative control experiments (*N* = 8 per genome) using non-targeting IgG or anti-HA antibodies. Both human and mouse blacklists show high concordance with these negative control experiments and identify > 80% of the by-definition artifactual peaks called. We tested the blacklists on published C&R datasets and show that they lead to identification of false positive peaks that often escape elimination when peak calling is performed against a negative control or when previous blacklists were applied. Lastly, we show that subtracting the C&R-specific blacklists before peak calling can improve the performance of both SEACR and MACS2 and the consequent reliability of C&R peaks.

## Results

### Generation of the suspect lists

**Fig. 1:**
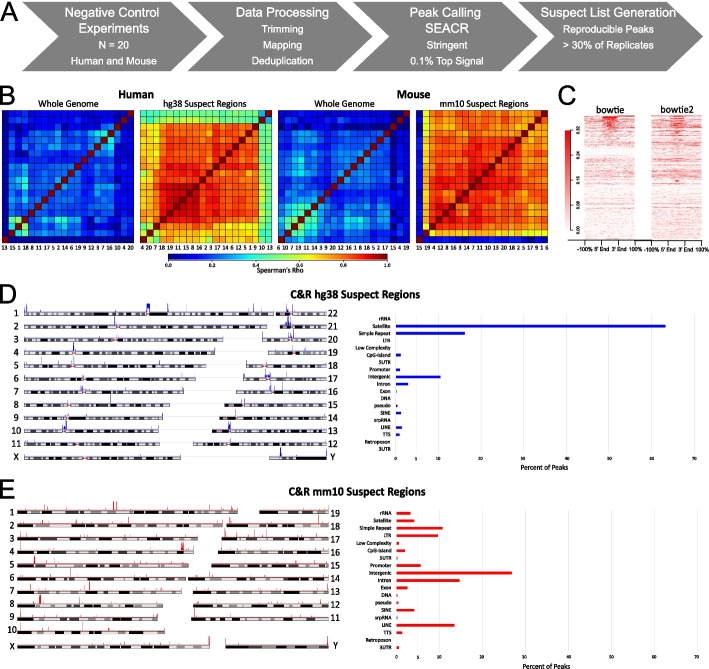
Generation of the CUT&RUN suspect lists. **A** Schematic representation of suspect list generation strategy. **B** Plots of Spearman’s correlation between negative control samples (*N* = 20 each) in human and mouse, showing genome-wide correlation and correlation within the C&R suspect list regions in hg38 and mm10 genomes. In both human and mouse, samples show dramatically increased correlation when considering only the suspect list regions. The mouse dataset (15) that does not correlate well with the rest can be explained by an unequal distribution of reads among the chromosomes, with high enrichment for chromosome 1. **C** Signal enrichment plots of the hg38 suspect list regions for a representative human negative control sample, after mapping with bowtie or bowtie2. More stringent mapping with bowtie did not drastically change the enrichment in the suspect listed regions. **D** Chromosome map showing genome wide locations of regions contained in the C&R hg38 suspect list (left) and corresponding genomic annotations by HOMER (right). The hg38 suspect list is enriched for satellite and simple repeat regions. **E** Chromosome map showing the genome wide location of regions contained in the C&R mm10 suspect list (left) and corresponding genomic annotations (right). The mm10 suspect list contains many different types of regions, the most represented being intergenic loci

We compiled human and mouse genomic C&R suspect lists by downloading publicly available raw C&R data from the European Nucleotide Archive (https://www.ebi.ac.uk/ena/browser/home) and processing them to identify high signal regions (Fig. 1A). We included 40 negative controls (20 for human and 20 for mouse, all with unique cell types) from 40 different studies to ensure diversity on the model systems used and identify cell-type agnostic regions. These datasets were produced with varying conditions, including different cell number, pA-MNase or pA/G-MNase, and control antibody type (full details in Additional file 1: Table S1). The criteria for inclusion were a clear labeling system as C&R negative controls (i.e., “no antibody,” “IgG,” etc.) and the use of pair-end NGS technology. We performed trimming on the data to remove adapters and then mapped to the human (hg38) or mouse (mm10) genomes with bowtie2, using commonly recommended settings for C&R data [9, 10]. Once mapped, we deduplicated the BAM files to stringently identify artifacts that occur regardless of PCR duplication rates. We applied peak calling of these negative control samples with SEACR on stringent mode, using a threshold of 0.001 to identify the highest 0.1% signals. We then extended the peak regions by 1000 bp in either direction, to ensure capturing peaks that may be slightly shifted between datasets and overlapped the peak sets resulting from each different individual sample to measure the reproducibility of false positive signal. The suspect lists are compiled by peaks present in at least 30% of the negative controls used: we consider this probability sufficiently high to indicate an unacceptable chance of false positive hits. The suspect lists are provided in Additional file 2. When peak numbers are plotted based on the number of datasets in which they are found, 7 of 20 is located at the approximate “knee” of the curve, where the reproducibility begins to stabilize (Additional file 1: Figure S1A).

Genome-wide, the datasets show very low correlation (Fig. 1B), which is expected due to random distribution of MNase-based digestion when a non-targeting antibody is used [9]. However, when considering only the suspect listed regions the correlation is high (Fig. 1B), indicating that these regions are enriched across independent negative control samples and ought to be considered as artifactual. To ensure that the suspect lists are valid regardless of the data processing pipeline, we applied more stringent mapping with bowtie—instead of bowtie2—allowing neither sequence mismatches nor multimapping. Though the fraction of reads within the suspect list peaks (FRIP score; a typical measure of the efficiency of the experiment) typically decreased with more stringent mapping (Additional file 1: Table S2), the average signal enrichment profile across suspect list regions was comparable and still enriched compared to background (Fig. 1C), demonstrating that a majority of these problematic regions display real signal enrichment independently from mapping biases.

The suspect listed regions are scattered throughout the genome, both in human and in mouse, and involve all chromosomes (Fig. 1D and E, respectively, left panels). The C&R suspect lists also contain the mitochondrial genome, as well as regions from unknown or random chromosomes which are mappable with bowtie2, as these regions can contribute to noise in experiments. The regions in both lists have an average size around 5000 bp. We checked the mappability in the suspect listed regions using UMAP [11] and found that 656/1049 regions in hg38 and 42/559 in mm10 are considered lowly mappable by 36 bp reads. When annotating the suspect lists for genomic regions, the hg38 suspect list is dominated by satellite and simple repeat regions, though it also contains regions annotated in other categories like intergenic, intronic, and promoter regions (Fig. 1D, right). The mm10 suspect list has a more even distribution of region annotations, with the most falling in intergenic regions (Fig. 1E, right). Fully annotated versions of the suspect lists, including the region coordinates, annotation, gene annotation to the closest TSS, mappability, and overlap with the ENCODE lists, are provided in Additional file 2.

### Comparison of the CUT&RUN suspect lists with the ENCODE blacklists

**Fig. 2:**
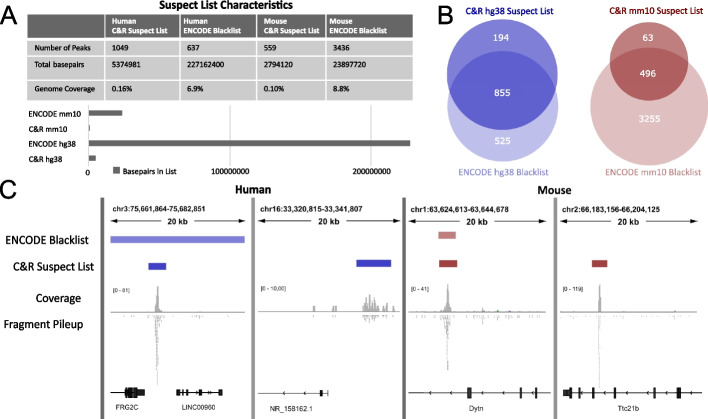
Comparison of the CUT&RUN and ENCODE suspect lists. **A** Comparison of number of peaks, base pairs suspect listed, and total genome coverage for C&R and ENCODE hg38 and mm10 suspect lists. The C&R suspect lists cover less of the genome compared to their ENCODE counterparts. **B** Comparison of overlapping suspect listed regions between C&R and ENCODE hg38 and mm10 suspect lists, showing both considerable overlap and unique regions. **C** Examples of coverage and fragment pileups for representative negative controls tracks in human and mouse, showing both unique regions for the C&R suspect lists and regions shared with ENCODE. C&R = CUT&RUN

When compared with the latest version (v2) of the ENCODE blacklists [6], our C&R suspect lists cover a smaller portion of the genome (Fig. 2A). For the comparison, we manually added the mitochondrial genome to the ENCODE blacklists, as this is recommended to remove but not included in the list. Several ENCODE blacklist regions are broader than the C&R suspect list regions; the ENCODE blacklisted regions were in fact extended to encompass unmappable or neighboring high-signal regions, and blacklist regions within a certain distance range were merged [6]. In contrast, we chose not to manually curate or merge the identified regions, or extend them beyond 1000 bp in either direction, to provide the dual advantage of (i) increasing the specificity in identifying the high-signal C&R false peaks and (ii) removing as little of the genome as possible. Therefore, one ENCODE blacklist peak may overlap with multiple C&R suspect peaks. While due to their different biochemistries ChIP-seq and C&R can produce different sets of false positives, we noticed that several false peaks are shared between the ENCODE and C&R suspect lists (Fig. 2B). We interpret that these artifacts could be caused either by undesired enrichment of prevalent fragments in the genome, such as repeated sequences, or by genome assembly and mapping biases [6]. Moreover, the overlap between the ENCODE and the C&R suspect lists constitutes an important validation of our approach. On the other hand, as expected, there are C&R specific regions absent in the ENCODE lists (Fig. 2C), and many regions included in the ENCODE blacklists do not show any signal enrichment in the C&R negative controls. Thus, employing the ENCODE blacklists to filter C&R data might lead to both the undesired consequences of unnecessarily removing regions of true signal and failing to remove C&R-specific artifacts.

Improved assemblies have recently been released for human (telomere-to-telomere, T2T) and mouse (mm39). In the future, researchers will likely move towards mapping to these genomes instead of hg38 and mm10, so we compiled suspect lists with the same method for these new assemblies and included them in Additional file 2. While the mm39 suspect list contains a comparable number of regions to the mm10 dedicated one, the T2T suspect list is made by 212 regions versus the 1049 for hg38. This likely represents the increased confidence of the more recent genome assembly and indicates that some artifacts commonly found in C&R data are indeed computational in nature.

### Experimental validation of the CUT&RUN suspect lists

**Fig. 3:**
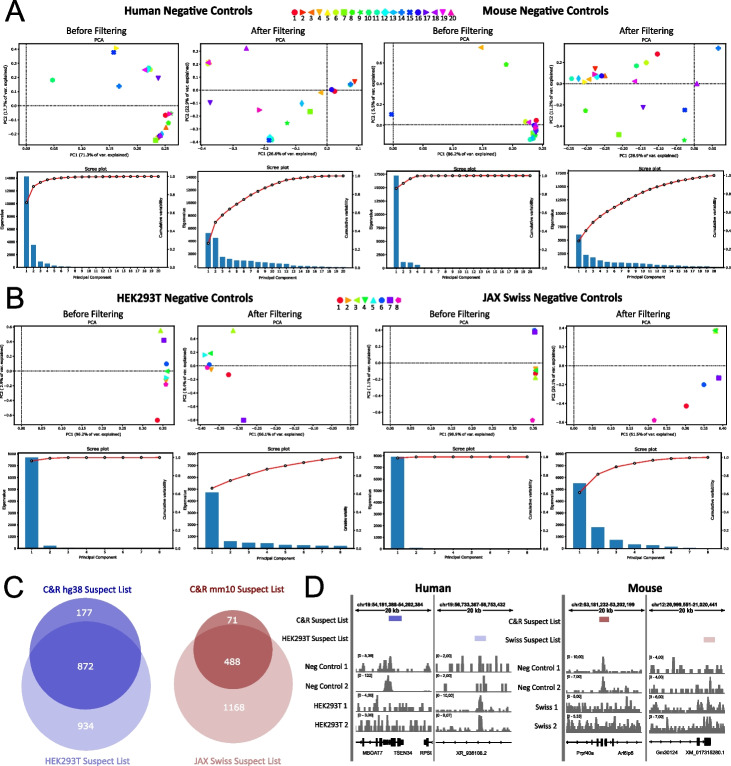
Experimental validation of the CUT&RUN suspect lists. **A** Principal component analysis graphs (top row) and Scree plots (bottom row) of human and mouse negative control samples, showing a decrease in sample similarity after filtering with C&R suspect lists. The percentage of variance explained by each principal component decreases, and the samples become more randomly distributed on the graphs, indicating more variance in line with an expected random distribution of reads from a non-specific antibody in C&R. The Scree plots show a decrease in the Eigenvalue and contribution of principal component 1 to the total observed variance after filtering. **B** Principal component analysis (top row) and Scree plots (bottom row) on human and mouse negative controls performed to validate the C&R suspect lists, showing increase variance after filtering. **C** Comparison of C&R suspect lists with suspect lists built with the same method based on experiments performed in HEK293T human cells and embryonic tissues from JAX Swiss mice. The suspect lists show high concordance with over 80% of each C&R suspect list being identified by the cell or strain specific suspect lists. **D** Examples of genome coverage of representative samples under unique regions of the C&R and cell/strain specific suspect lists. C&R, CUT&RUN; PCA, principal component analysis

To validate the C&R suspect lists, we first turned to the negative controls they were built on. Principal component analysis (PCA) plots of the genome-wide signal distribution revealed a reduction in sample similarity after filtering with the suspect lists, both for human and mouse C&R datasets (Fig. 3A, top panels). Scree plots for each PCA show that subtracting the suspect lists decreases the Eigenvalue of principal component 1 and increases the contribution of the other principal components on the total variance observed (Fig. 3A, bottom panels). This indicated that the C&R suspect lists successfully identify and subtract the commonalities across negative control sample, which by definition should be considered false positives. In addition, to test our C&R suspect lists experimentally, we decided to carry out a series of new experiments: we performed 8 C&R tests in HEK293T human cells and 8 in mouse embryonic tissues from JAX Swiss mice by using either IgG or anti-HA antibodies. To increase diversity in this test, we used both the original C&R protocol [1] and our recently developed C&R-LoV-U version for non-DNA-binding transcriptional co-factors [12]. A full list of sample information is provided in Table 2. The obtained datasets were analyzed as shown in Fig. 1A to replicate the construction of the suspect lists but solely built on these new data. The C&R suspect lists were able to increase the variance in our internally generated negative controls (Fig. 3B). Most importantly, our new datasets contained > 80% of the regions identified by each C&R suspect list (Fig. 3C), indicating that the C&R suspect lists (i) contain reproducibly enriched regions, (ii) are generally applicable, and (iii) do not contain any obvious cell type-specific bias. In our opinion, the additional peaks of our internally generated series of negative controls not found by the suspect lists are likely due to HEK293- or mouse strain-specific enrichment (Fig. 3D). This reinforces the need for performing negative controls at each experiment, in addition to using our suspect lists.

We were curious as to whether identifiable factors, such as type of negative control, pA- or pA/G-MNase, or C&R protocol adopted could influence the pattern of signal. To investigate this, we marked the samples in the PCA plots in different colors based on these factors and followed their behavior before and after filtering for the datasets used to build the suspect lists (Additional file 1: Figure S1B) and our own in-house generated negative controls (Additional file 1: Figure S1C) to determine the existence of any clustering pattern. From this, confirming our experience, there does not seem to be discernible patterns in signal due to these factors. Regardless of conditions, we noticed that when the data from the control is sparser (datasets 4, 7, 13, and 20 in Fig. 1B which are less correlated with the rest), it renders it more difficult to identify artifacts. In these cases, suspect lists are particularly well-suited to assist removing artifact regions that are not enriched in the negative control due to low coverage.

### Filtering via CUT&RUN suspect lists improves peak signal intensity of published datasets

**Fig. 4:**
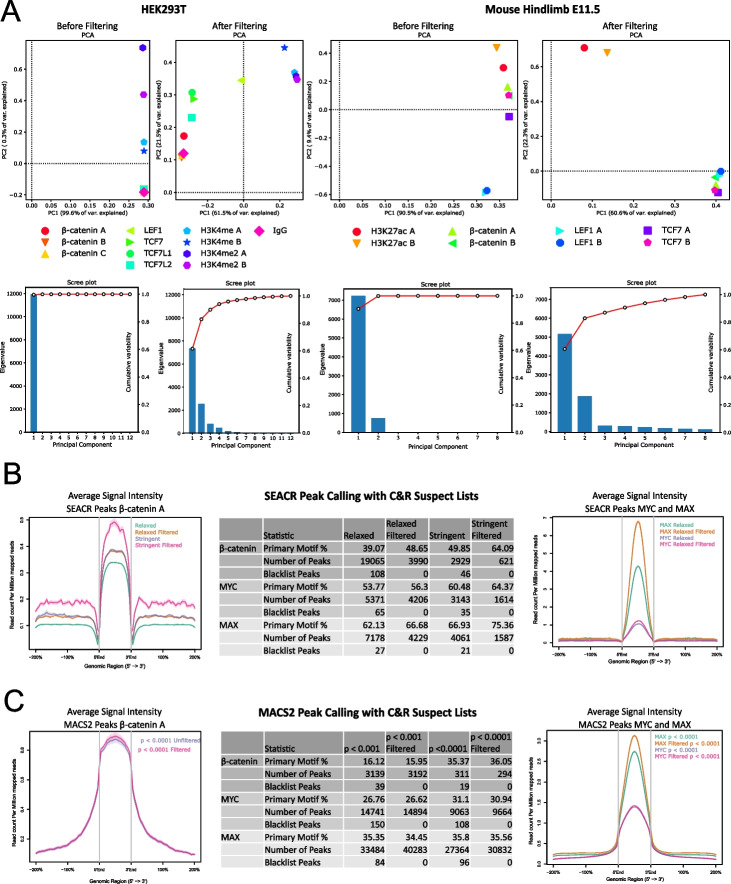
Application of the CUT&RUN suspect lists on published datasets. **A** Principal component analysis on published C&R datasets (top row) and Scree plots (bottom row) from [11], showing that suspect list filtering reduces sample similarity and allows samples to clustering as expected based on the antibody used. This was confirmed by calculation of the average ratio of inter-target versus intra-target distance, which increased with filtering from 2.68:1 to 4.73:1 in HEK293T and from 2.18:1 to 8.53:1 in the mouse hindlimb. **B** Evaluation of suspect list filtering on SEACR peak calling. Left: Comparison of peaks called using SEACR against a negative control on relaxed and stringent modes for the β-catenin A replicate, showing that suspect list filtering of BAM files before peak calling leads to a smaller number of called peaks which have an increased average signal profile within the peaks and an increased percentage of peaks which contain expected TCF/LEF motifs (middle). The increase in background signal in the filtered and more stringent sets in the average profile (left) is due to an increase in peaks called in high-signal regions, not suspect list filtering (see Supplementary Fig. 1D). Right: Comparison of peaks called by SEACR on MYC and MAX datasets from [1] before and after suspect list filtering, showing a decrease in peak number accompanied by an increase in average signal within the peak regions. **C** Evaluation of suspect list filtering on MACS2 peak calling. Left: calling β-catenin peaks with MACS2 after filtering leads to peaks with a comparable but slightly higher average signal profile. Center: Peak calling with MACS2 on MYC and MAX at two different stringencies before and after filtering shows that suspect list regions are called as peaks in unfiltered sets and that filtered sets contain more peaks at the same statistical stringency. Right: The average profiles of filtered MYC and MAX peak sets show a higher signal to noise ratio than their unfiltered counterparts. C&R, CUT&RUN; PCA, principal component analysis

We set out to evaluate the performance of the C&R suspect list on published C&R datasets. First, we performed PCA analysis before and after suspect list filtering on human and mouse C&R-LoV-U datasets obtained across different types of targets, histone modifications, transcription factors, and co-factors [12, 13]. This invariably led to similar improvements: first, the genome-wide variance of the signal increased; second, the samples clustered more, as expected, with their replicates or DNA binding partners. These indicate that the suspect list regions were falsely inflating the similarities of the samples (Fig. 4A). This was confirmed by calculation of average intra-target versus inter-target distances: intra-target distances decreased after filtering (suggesting increased experimental/analytical precision), while inter-target distances increased (indicating specificity, that is the identification of biologically meaningful differences depending on the selected target). The ratio of the average inter-target versus intra-target distance increased from 2.68:1 to 4.73:1 in HEK293T, and from 2.18:1 to 8.53:1 in the hindlimb datasets upon suspect list filtering. We consider these as quantitative measures of the improvement in the datasets. We noticed that some of the suspect listed regions even appeared, in the published description of this experiment, within the list of “high-confidence” peaks for β-catenin in both human cells and mouse tissue, despite that these lists were curated by the subtraction of regions enriched in the negative control and of the ENCODE blacklists, enforcing the need for C&R specific suspect lists.

SEACR was benchmarked on datasets without removing high-signal regions and often performs well as is on high-efficiency experiments. However, we hypothesized that signal within suspect list regions could be affecting the peak calling algorithm, both in the normalization of sample to control and in the later determination of signal thresholds. We tested SEACR on the β-catenin data with both relaxed and stringent settings and show that removing all the reads mapping to the C&R suspect listed regions before peak calling results in smaller, yet more reliable peak sets with fewer false positives. We conclude this based on the measurement of two key parameters: (i) a higher enrichment for the expected primary motif and (ii) an improved average signal profile in the peaks (Fig. 4B, left and center). To test this further, we downloaded the raw data for MAX and MYC chromatin profiling performed in human K562 cells, along with the no-antibody negative control, from the original C&R publication [1, 14]. First, we mapped these datasets with bowtie2 to the hg38 genome with and without deduplication, then calculated FRIP scores for the C&R hg38 suspect list. The MYC and no-antibody datasets displayed enrichment for the suspect list regions, with FRIP scores ranging from 5 to 8%. The MAX dataset was especially affected by the suspect list regions: it had 71.9% of fragments within the suspect list peaks before deduplication, and 21.6% after duplicate removal. When called with SEACR against the negative control, before and after suspect list filtering, we detected a reduction in peak number for both MYC and MAX, an increased average signal-to-noise ratio within the peaks (Fig. 4B, center and right) and an increased presence of the primary motif. This difference is especially strong for MAX, which had the higher FRIP score, and thus had likely a greater effect from signal within the suspect list regions. These analyses indicate that suspect list filtering of bam files before peak calling has a larger impact on the final results when compared to filtering after peak calling with SEACR: the number of regions removed was much higher than the number of suspect list regions present in each unfiltered peak set (Fig. 4B, center), confirming that the performance of SEACR is affected by the spurious signal in suspect list regions.

Interestingly, analogous suspect list filtering also affects peak calling executed with MACS2. With MACS2, calling the unfiltered β-catenin dataset against the control resulted in suspect list regions being called as peaks (Fig. 4C, center), showing that even though MACS2 uses local background, artifact peaks can still “slip through.” Additionally, the filtered dataset had slightly more peaks called at the same stringency once suspect list peaks were retrospectively removed (294 versus 292). When compared with average profiles, the signal per million reads was higher in the filtered set, likely because of reduced total signal after removal of suspect list reads (Fig. 4C, left). Next, we tested MACS2 peak calling for the MYC and MAX datasets and saw the same general pattern of suspect list regions being called in the unfiltered datasets, at two stringencies, and more peaks with a stronger average profile in the filtered datasets (Fig. 4C, middle and right). The presence of the primary motif was not consistently affected by filtering, with the difference being less than 1% in all cases, sometimes lower or higher after filtering (Fig. 4C, center). While these effects are not as drastic as we observed with SEACR, these analyses show that suspect list filtering of C&R processed with MACS2 is advantageous, whether the filtering is performed before or after the peak calling.

## Discussion

Proper controls are vital in any type of experiment, and C&R is no exception. In C&R, the primary goal of a negative control is to identify regions which become enriched due to factors other than the physical occupancy of a protein target (artifacts), so that they may be removed and prevented from confounding any subsequent analyses on the primary target’s binding pattern. The nature of the signal enrichment in the artifact regions and the mechanisms by which they appear are by no means solved by this analysis. The existence of C&R specific artifacts, not found in ChIP-seq blacklists, and artifacts specific to a certain cell type or mouse strain, point towards a biological origin. On the other hand, the lower number of regions in our C&R suspect list for the T2T assembly versus hg38 indicates that a majority of these could be computational, as they have been resolved by a more complete reference genome sequence. Repeat regions are common in the genome and thus could naturally be enriched in background DNA. Library preparation PCR could amplify them even further, and finally an imperfect genome assembly could erroneously map them in one region, thereby creating an artifactual enrichment. In these cases, the explanation for their existence would be a combination of multiple factors. The differences between where suspect list regions are located in hg38 (primarily satellite regions) versus mm10 (more equally distributed, many in intergenic and intronic regions) are also interesting and make it difficult to generalize about the nature of these regions and translate the findings to other organisms (as it could reflect species-specific idiosyncrasies) or genome assemblies. Moreover, in both suspect lists generated, there is an overlap with expected real biological signal regions, such as promoters or intronic regions. This means there is the possibility that filtering could remove real signal; while this should be avoided, if possible, we generally feel it is best to err on the side of caution and risk false negatives as a cost for reducing false positives.

In addition to generating C&R specific suspect lists containing commonly enriched artifact regions, we also tried to glean information about the best control design from our analysis. From our search, the most commonly used type of negative control seems to be IgG; other options include epitope-tag antibodies that model a knockout situation where the epitope is not present (e.g., anti-HA, anti-GFP) or no-antibody controls. Interestingly, we did not detect major differences in the coverage or artifact recognition by different types of antibodies nor did we see differences due to the protocol or type of enzyme used. However, we did notice that negative controls do not always show enrichment in all artifact regions and that this is more prone to happen on a larger scale when the genome coverage is very sparse. This could be due to a lack of sequencing depth in the negative control, or from an extremely “clean” experiment where very little DNA is recovered, thus resulting in a low complexity library with a majority of reads filtered out after deduplication. In these situations, the C&R suspect lists will be an especially useful tool. However, we suggest that the suspect lists generated here should, when possible, accompany rather than replace adequate negative controls performed in the same conditions as the test samples; our internally generated suspect lists point to the existence of cell-type specific artifacts which cannot be fully accounted for by a universal suspect list.

Even if all artifactual regions were to be equally present in a negative control, we show that they can still be erroneously called as peaks and affect the peak calling algorithms themselves. We tested on the most commonly used peak callers for C&R: SEACR and MACS2, but this likely holds true for others. When SEACR is performing well and yields results compatible with current knowledge of the specific factor, it may not be needed to filter out suspect list regions before peak calling or it may increase stringency to the point of excluding real peaks. However, when it calls very few or excessive numbers of peaks, we find that filtering can significantly improve the final outcome. This implies that SEACR, which is based on the detection of enrichment regions that stand out against the global background, is particularly sensitive to spurious signal. MACS2 is often better at not calling artifactual peaks due to using local versus global background; however, we show that suspect list filtering prior to peak calling improves the performance of the peak algorithm in the datasets tested and that filtering after peak calling does in almost all cases remove artifactual peaks that slipped past the high-stringency standards of MACS2. We submit that removal of the C&R suspect list, whether before or after peak calling and regardless of the peak caller used, is a useful tool to increase reliability of peak sets.

To generate the suspect lists, we parsed through approximately 200 projects on the ENA to find independent C&R datasets performed on human and mouse tissues and used 20 each to assemble a C&R suspect list of problematic regions. In searching for data to use, we came across many deposited experiments that either completely lacked negative controls, or did not possess a clear sample labeling system, preventing us from their inclusion. At this moment, these C&R suspect lists are built on comparably less data than others previously published lists (e.g., the ENCODE Blacklist), and this might limit its robustness and applicability. We submit that initiatives aimed at improving the clarity of complete data deposition is an important step for the generation of future suspect lists. However, with the diversity of data used to build the suspect lists and their subsequent successful validation, we believe we have compiled a mature selection of artifact regions that are a typical by-product of C&R experiments and are cell-type agnostic. We cannot conclusively exclude that our suspect lists contain some regions that could be cell-type or strain specific or fail in identifying additional problematic regions that would appear in other cell types. Nevertheless, in the light of the many publicly available datasets lacking negative controls, we believe that our suspect list is an essential tool.

## Conclusions

Using publicly available C&R negative control data, we have compiled suspect lists (for the hg38 and T2T human genomes, and mm10 and mm39 mouse genomes) containing artifact regions that are consistently and spuriously enriched across experiments. We have shown that some artifact regions are unique to C&R, indicating the need for technique-specific suspect lists and implying a partially biological origin to the signal enrichment, while the reduction in number of regions for the improved genomic assemblies implies a computational nature. We validated our suspect lists both by using reiterative negative control experiments and by applying them to published datasets, showing that suspect list filtering improves the confidence of peak datasets obtained with SEACR and MACS2. Future work is needed to compile similar suspect lists for other model organisms as C&R becomes more widely used in other fields of research.

## Material and methods

### Suspect list generation

Publicly available C&R data are as follows: 40 negative controls (20 for human and 20 for mouse) were downloaded from the ENA archive; a full list of accession and references are in Table 1. Read trimming was performed using bbmap bbduk [15] (version 38.18) removing adapters, artifacts, and poly AT, TA, G and C repeats. Alignment was done to the hg38 genome or mm10 genome using bowtie2 (version 2.4.5) [16], settings included –local –very-sensitive-local –no-unal –no-mixed –no-discordant –dovetail –X 700. For bowtie [17] (version 1.0.0), we used the options -v 0 -m 1 -X 500. Samtools [18] (version 1.11) was used to create bam files, mark and remove duplicates when applicable, and sort bam files. Bedgraphs were created using bedtools [19] (version 2.23.0), genomecov with pair-end settings. Peaks were called using SEACR [8] (version 1.3) for each bedgraph using the settings norm and stringent with a threshold set to 0.001. Peak regions were extended using bedtools slop to 1000 bp in either direction from the peak. Bedsect [20] was used to overlap peak regions on default settings, and peaks called in greater than 30% of negative controls (> = 7 of 20) were kept. Bedtools merge and sort was used to combine overlapping peaks to generate the final suspect list.

**Table 1:** CUT&RUN public data

**Human**				**Mouse**			
**Number**	**Project**	**Sample**	**Reference**	**Number**	**Project**	**Sample**	**Reference**
1	PRJNA758691	SRR15663649	[21]	1	PRJNA682243	SRR13188236	[22]
2	PRJNA753322	SRR15403020	[23]	2	PRJEB41862	ERR4973502	[24]
3	PRJNA699604	SRR13634070	[25]	3	PRJNA777234	SRR16694279	[26]
4	PRJNA655230	SRR12387746	[27]	4	PRJNA744774	SRR15069561	[28]
5	PRJNA427801	SRR6426139	[29]	5	PRJNA719369	SRR14134905	[30]
6	PRJNA776721	SRR16674836	[31]	6	PRJEB51482	ERR9130874	[32]
7	PRJNA756549	SRR15539083	[33]	7	PRJNA746301	SRR15123732	[34]
8	PRJNA721947	SRR14238385	[35]	8	PRJNA722185	SRR14243166	[36]
9	PRJNA691366	SRR13586925	[37]	9	PRJNA753786	SRR15414824	[38]
10	PRJNA816825	SRR18337644	[39]	10	PRJNA744230	SRR15054366	[40]
11	PRJEB55317	ERR10047705	[41]	11	PRJNA786482	SRR17139040	[42]
12	PRJNA836267	SRR19139489	[43]	12	PRJNA860380	SRR20325067	[44]
13	PRJNA798000	SRR17642967	[45]	13	PRJNA656290	SRR12424468	[46]
14	PRJNA717224	SRR14068376	[47]	14	PRJNA862741	SRR20665931	[48]
15	PRJNA704964	SRR13785931	[49]	15	PRJNA658977	SRR12507586	[50]
16	PRJNA682426	SRR13194196	[51]	16	PRJNA678949	SRR13073031	[52]
17	PRJNA888075	SRR21837879	[53]	17	PRJNA527826	SRR8745654	[54]
18	PRJNA413473	SRR6144305	[55]	18	PRJNA682340	SRR13190129	[56]
19	PRJNA647352	SRR12267593	[57]	19	PRJNA864644	SRR20727958	[58]
20	PRJNA562266	SRR10022374	[59]	20	PRJNA493794	SRR7939979	[60]

### Graphs and analysis

deepTools [61] (version 3.5.1–0) multiBamSummary on bins mode for whole-genome, and bed mode using the suspect list bed file, followed by plotCorrelation were used to create correlation heatmaps with Spearman’s correlation. HOMER [62] annotatePeaks on default settings was used to determine genome annotation categories. FRIP scores were calculated from total number of fragments mapping to suspect listed peaks, divided by total number of mapped fragments. Signal intensity plots and average profiles were created using ngsplot [63] (version 2.63) with the settings -G hg38 -R bed -N 2 -SC global -IN 0. The UCSC genome browser was used to create the chromosome graph [64]. Intervene [65] (version 0.6.4) was used to overlap peak sets and create Venn diagrams. IGV [66] was used for bedgraph and bed file visualization. The ENCODE v2 blacklists were downloaded from [6], and the mitochondrial genome was manually added for a fair comparison.

### Suspect list validation on published data

Datasets performed by [12] were downloaded from [13]. Human and mouse data from Zambanini et al. [12] were processed as previously described in the paper. Raw data for MAX, MYC, and no antibody negative control in K562 cells from [1] were downloaded from [14] and were processed with bowtie2 as previously described, with or without removal of duplicates. Genome wide PCA plots and accompanying Scree plots were generated by first using deepTools multiBamSummary as described above on original data and by using -bl to filter out the C&R suspect lists, and then the graphs made with plotPCA with default settings. Suspect list filtering prior to peak calling was performed on the BAM files with bedtools intersect -v. Peak calling was performed with SEACR against the negative control with the settings norm and relaxed or norm and stringent. With MACS2 [67] peak calling was performed against the negative control with option -f BAMPE against the negative control. Signal profile graphs were created with ngsplots with the settings -G hg38 -R bed -N 2 -SC global -IN 0. Motif analysis was done using HOMER [62] (version 4.11) findMotifsGenome with the -size given option.

### CUT&RUN experiments

Human negative control data was obtained from experiments on HEK293T cells. Cells were cultured at 37 °C in a humidified incubator with 5% CO_2_ in high glucose Dulbecco’s Modified Eagle Medium (Cat. #41965039, Gibco) supplemented with 10% bovine calf serum (Cat. #1233C, Sigma-Aldrich) and 1X Penicillin–Streptomycin (Cat. #15140148, Gibco). Cells were stimulated with 10 μM CHIR99021 (Cat. #SML1046, Sigma Aldrich), 1 nM LGK (Cat. # S7143, Selleck Chemicals), or no stimulation. Mouse negative control data was obtained from various tissues from JAX Swiss Outbred mice (strain #: 034608) embryos at 11.5 dpc. Animal experimentation and housing conditions were according to the Swedish laws and guidelines under and performed under the ethical animal work license obtained by C.C. at Jordbruksverket (Dnr 2456–2019). Tissue dissociation, cell harvest, CUT&RUN or CUT&RUN LoV-U, and library preparation were performed as described in [12], CUT&RUN based on the original method by [1]. Anti-IgG (ABIN101961) or anti-HA (05-902R, Merck Millipore) antibodies were used. Samples were sequenced 36 bp pair-end on the NextSeq 550 (Illumina) using the Illumina NextSeq 500/550 High Output Kit v2.5 (75 cycles) (Cat. #20024906, Illumina). Sample information provided in Table 2.

**Table 2:** C&R experiment sample information

**HEK293T**				**JAX Swiss**			
**Number**	**Condition**	**Protocol**	**Antibody**	**Number**	**Tissue**	**Protocol**	**Antibody**
**1**	None	C&R	HA	**1**	Hindlimb	C&R LoV-U	IgG
**2**	Chir 4 h	C&R LoV-U	HA	**2**	Forelimb	C&R	IgG
**3**	Chir 24 h	C&R LoV-U	IgG	**3**	Branchial arches	C&R	IgG
**4**	LGK	C&R	HA	**4**	Heart	C&R	IgG
**5**	Chir 24 h	C&R	HA	**5**	Hindlimb	C&R	IgG
**6**	Chir 24 h	C&R	IgG	**6**	Forelimb	C&R LoV-U	IgG
**7**	Chir 24 h	C&R LoV-U	IgG	**7**	Branchial arches	C&R LoV-U	IgG
**8**	Chir 4 h	C&R LoV-U	IgG	**8**	Forelimb	C&R	HA

### Supplementary Information

**Additional file 1: Figure S1.** A. Line graphs display the number of peaks (y-axis) and number of datasets (x-axis) showing the reproducibility of SEACR called peaks in the negative control datasets used to build the suspect lists for hg38 (left) and mm10 (right). The grey dot at 7, at the approximate “knee” of the curve, represents the threshold chosen for the suspect list compilation. B. PCA plots of negative control datasets used to compile the suspect lists before and after suspect list filtering, colored by antibody used (top; IgG in blue, “others” in pink) and protein used (bottom; pA/G-MNase in blue, pA-MNase in pink). C. PCA plots of internally generated negative control datasets colored by antibody used (top; IgG in blue, anti-HA in pink) and protocol used (bottom; C&R in blue, C&R LoV-U in pink). D. Alternate average signal intensity plot of β-catenin before and after filtering, after peaks in regions of high overall signal due to genomic duplications were removed. The smaller (filtered stringent) datasets were most affected by these regions, and this was the cause of the inflated background signal in the plot in Fig. 4A. C&R = CUT&RUN, C&R LoV-U = CUT&RUN Low Volume Urea, PCA = Principal Component Analysis. **Table S1.** Reference and experimental information about datasets used in the complication of C&R suspect lists. **Table S2.** Fragments within Peaks (FRIP) scores for C&R blacklist regions within negative control samples after mapping with bowtie2 or bowtie.

**Additional file 2.** Suspect list genome coordinates for hg38, mm10, T2T, mm39, and coordinates with annotations for the hg38 and mm10 lists.

**Additional file 3.** Review history.

## Data Availability

Publicly available datasets used in this study were accessed via the ENA and can be downloaded from the short read archives (SRA) according to their accession information: ERP139584 [13], SRP078609 [14], SRR15663649 [21], SRR13188236 [22], SRR15403020 [23], ERR4973502 [24], SRR13634070 [25], SRR16694279 [26], SRR12387746 [27], SRR15069561 [28], SRR6426139 [29], SRR14134905 [30], SRR16674836 [31], ERR9130874 [32], SRR15539083 [33] SRR15123732 [34], SRR14238385 [35], SRR14243166 [36], SRR13586925 [37], SRR15414824 [38], SRR18337644 [39], SRR15054366 [40], ERR10047705 [41], SRR17139040 [42], SRR19139489 [43], SRR20325067 [44], SRR17642967 [45], SRR12424468 [46], SRR14068376 [47], SRR20665931 [48], SRR13785931 [49], SRR12507586 [50], SRR13194196 [51], SRR13073031 [52], SRR21837879 [53], SRR8745654 [54], SRR6144305 [55], SRR13190129 [56], SRR12267593 [57], SRR20727958 [58], SRR10022374 [59], and SRR7939979 [60]. C&R negative control experiments performed in this study have been deposited at ArrayExpress, E-MTAB-12411 (https://www.ebi.ac.uk/biostudies/arrayexpress/studies/E-MTAB-12411?accession=E-MTAB-12411), and are publicly available. The C&R suspect lists for hg38, T2T, mm10 and mm39 are provided in Additional file 2.
