# Supplementary material for: The CUT&RUN suspect list of problematic regions of the genome
Source: Genome Biol. 2023 Aug 10;24:185. doi: 10.1186/s13059-023-03027-3 (PMC10416431; doi:10.1186/s13059-023-03027-3)
Supplement: Supplementary file 1 — Additional file 1: Figure S1. A. Line graphs display the number of peaks (y-axis) and number of datasets (x-axis) showing the reproducibility of SEACR called peaks in the negative control datasets used to build the suspect lists for hg38 (left) and mm10 (right). The grey dot at 7, at the approximate “knee” of the curve, represents the threshold chosen for the suspect list compilation. B. PCA plots of negative control datasets used to compile the suspect lists before and after suspect list filtering, colored by antibody used (top; IgG in blue, “others” in pink) and protein used (bottom; pA/G-MNase in blue, pA-MNase in pink). C. PCA plots of internally generated negative control datasets colored by antibody used (top; IgG in blue, anti-HA in pink) and protocol used (bottom; C&R in blue, C&R LoV-U in pink). D. Alternate average signal intensity plot of β-catenin before and after filtering, after peaks in regions of high overall signal due to genomic duplications were removed. The smaller (filtered stringent) datasets were most affected by these regions, and this was the cause of the inflated background signal in the plot in Fig. 4A. C&R = CUT&RUN, C&R LoV-U = CUT&RUN Low Volume Urea, PCA = Principal Component Analysis. Table S1. Reference and experimental information about datasets used in the complication of C&R suspect lists. Table S2. Fragments within Peaks (FRIP) scores for C&R blacklist regions within negative control samples after mapping with bowtie2 or bowtie. [file 13059_2023_3027_MOESM1_ESM.pdf]

Figure S1: A. Line graphs display the number of peaks (y-axis) and number of datasets (x-axis) showing the reproducibility of SEACR called peaks in the negative control datasets used to build the suspect lists for hg38 (left) and mm10 (right). The grey dot at 7, at the approximate "knee" of the curve, represents the threshold chosen for the suspect list compilation. **B.** PCA plots of negative control datasets used to compile the suspect lists before and after suspect list filtering, colored by antibody used (top; IgG in blue, "others" in pink) and protein used (bottom; pA/G-MNase in blue, pA-MNase in pink). **C.** PCA plots of internally generated negative control datasets colored by antibody used (top; IgG in blue, anti-HA in pink) and protocol used (bottom; C&R in blue, C&R LoV-U in pink). **D.** Alternate average signal intensity plot of β-catenin before and after filtering, after peaks in regions of high overall signal due to genomic duplications were removed. The smaller (filtered stringent) datasets were most affected by these regions, and this was the cause of the inflated background signal in the plot in Figure 4A. C&R = CUT&RUN, C&R LoV-U = CUT&RUN Low Volume Urea, PCA = Principal Component Analysis.

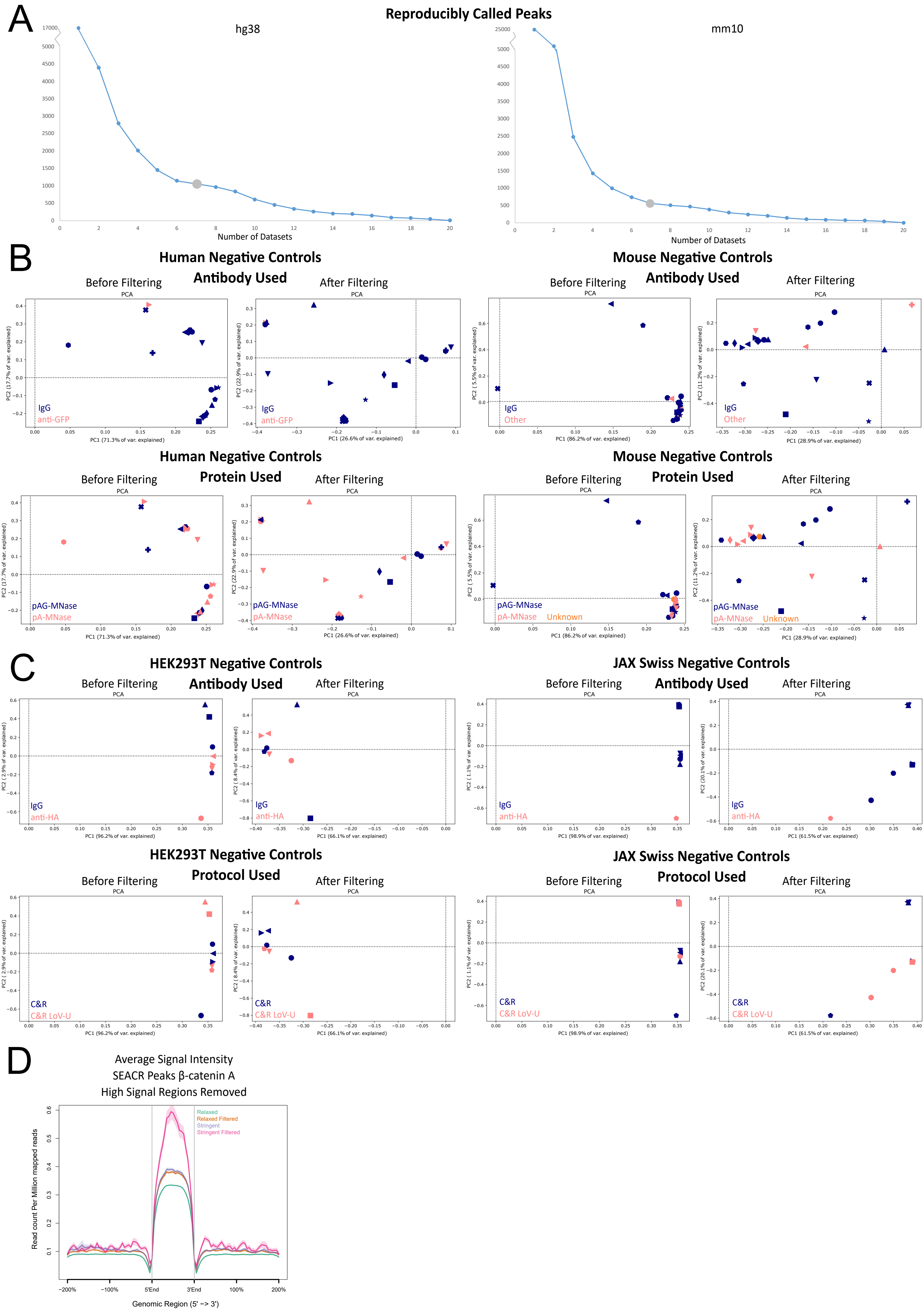
A
Reproducibly Called Peaks
hg38
Number of Datasets
mm10
Number of Datasets
B
Human Negative Controls
Antibody Used
Before Filtering
After Filtering
PCA
PC1 (71.3% of var. explained)
PC2 (17.7% of var. explained)
PC1 (26.6% of var. explained)
PC2 (22.9% of var. explained)
IgG
anti-GFP
Mouse Negative Controls
Antibody Used
PC1 (86.2% of var. explained)
PC2 (5.5% of var. explained)
PC1 (28.9% of var. explained)
PC2 (11.2% of var. explained)
IgG
Other
Human Negative Controls
Protein Used
pAG-MNase
pA-MNase
Mouse Negative Controls
Protein Used
pAG-MNase
pA-MNase
Unknown
C
HEK293T Negative Controls
Antibody Used
PC1 (96.2% of var. explained)
PC2 (2.9% of var. explained)
PC1 (66.1% of var. explained)
PC2 (8.4% of var. explained)
IgG
anti-HA
JAX Swiss Negative Controls
Antibody Used
PC1 (98.9% of var. explained)
PC2 (1.1% of var. explained)
PC1 (61.5% of var. explained)
PC2 (20.1% of var. explained)
HEK293T Negative Controls
Protocol Used
C&R
C&R LoV-U
JAX Swiss Negative Controls
Protocol Used
D
Average Signal Intensity
SEACR Peaks β-catenin A
High Signal Regions Removed
Relaxed
Relaxed Filtered
Stringent
Stringent Filtered
Read count Per Million mapped reads
-200%
-100%
5'End
3'End
100%
200%
Genomic Region (5' -> 3')

Table S1: Reference and experimental information about datasets used in the complication of C&R suspect lists

Human

Number	Project	Sample	Dataset Reference	Cell type	Cell #	Antibody	Protein	Protocol	Dup Rate	Mapped Reads
1	PRJNA758691	SRR15663649	[21]	Calu-3	1000000	IgG	pAG-MN	C&R	32.12	2642430
2	PRJNA753322	SRR15403020	[23]	SKMEL-1206	100000	IgG	pA-MN	C&R	18.19	4258320
3	PRJNA699604	SRR13634070	[25]	HUDEP-2		IgG	pA-MN	C&R	6.36	10543772
4	PRJNA655230	SRR12387746	[27]	HeLa	250000	IgG	pA-MN	C&R	87.77	624644
5	PRJNA427801	SRR6426139	[29]	Donor CD4+ T-Cells	6000000	antiGFP	pA-MN	C&R	1.08	1410722
6	PRJNA776721	SRR16674836	[31]	MKL-1		IgG	pA-MN	C&R	3.64	2565710
7	PRJNA756549	SRR15539083	[33]	CHLA-10		IgG	pAG-MN	Auto C&R	55.51	8724766
8	PRJNA721947	SRR14238385	[35]	ATAD-2 melanocytes	10000	IgG	pA-MN	C&R	11.67	5965576
9	PRJNA691366	SRR13586925	[37]	Human Islets	600000	IgG	pA-MN	C&R NE	16.4	987368
10	PRJNA816825	SRR18337644	[39]	RH30	200000	IgG	pA-MN	C&R	1.37	4452620
11	PRJEB55317	ERR10047705	[41]	hESC	500000	IgG	pAG-MN	C&R LoV-U	4.27	15785798
12	PRJNA836267	SRR19139489	[43]	Retina	200000	IgG	pA-MN	C&R	1.14	3921098
13	PRJNA798000	SRR17642967	[45]	ESC H9F	500000	IgG	pAG-MN	C&R	13.31	33750
14	PRJNA717224	SRR14068376	[47]	BE2C		IgG	pAG-MN	C&R	3.29	2860256
15	PRJNA704964	SRR13785931	[49]	Donor mDC		IgG	pAG-MN	C&R	10.06	30792658
16	PRJNA682426	SRR13194196	[51]	Bin67	200000	IgG	pAG-MN	C&R	37.4	36866814
17	PRJNA888075	SRR21837879	[53]	HEK293T	250000	IgG	pA-MN	C&R	5.7	22595546
18	PRJNA413473	SRR6144305	[55]	CD34	2000000	IgG	pA-MN	C&R NE	22.12	28931834
19	PRJNA647352	SRR12267593	[57]	CD4+ T cells	50000	IgG	pAG-MN	C&R	45.41	10521528
20	PRJNA562266	SRR10022374	[59]	SNU398	2000000	IgG	pA-MN	C&R NE	0.61	324712

Mouse

Number	Project	Sample	Dataset Reference	Cell type	Cell #	Antibody	Protein	Protocol	Dup Rate	Mapped Reads
1	PRJNA682243	SRR13188236	[22]	mESC		IgG	pAG-MN	C&R	5.43	3332006
2	PRJEB41862	ERR4973502	[24]	Neurospheres	100000	antiHA	pA-MN	C&R	11.93	11558730
3	PRJNA777234	SRR16694279	[26]	Oocytes	50000	IgG	pA-MN	C&R	25.46	9950696
4	PRJNA744774	SRR15069561	[28]	CD8 T cells		IgG	pAG-MN	C&R	60.73	4904222
5	PRJNA719369	SRR14134905	[30]	Primary erythroblasts	100000	IgG	pA-MN	C&R	12.26	4076668
6	PRJEB51482	ERR9130874	[32]	46C mESC		IgG	pAG-MN	C&R	11.94	1184342
7	PRJNA746301	SRR15123732	[34]	Intestinal epithelial	100000	IgG	pAG-MN	C&R	42.07	17388956
8	PRJNA722185	SRR14243166	[36]	Splenic B-cells	2000000 -	IgG	pAG-MN	C&R NE	6.34	6648740
9	PRJNA753786	SRR15414824	[38]	E10.5 yolk sac	15000	IgG	pAG-MN	C&R	74.34	1572348
10	PRJNA744230	SRR15054366	[40]	Spermatogenic cells	250000	IgG	pAG-MN	C&R	54.5	10775578
11	PRJNA786482	SRR17139040	[42]	Thymus CD4+	500000	IgG	pAG-MN	C&R	20.9	38024912
12	PRJNA860380	SRR20325067	[44]	Hippocampal neurons		IgG	pAG-MN	C&R NE	16.07	16159540
13	PRJNA656290	SRR12424468	[46]	C57BL/6 ESC		IgG	pA-MN	C&R	9.54	12215630
14	PRJNA862741	SRR20665931	[48]	CD1 intestinal epithelium		No ab	pAG-MN	C&R	26.57	37334796
15	PRJNA658977	SRR12507586	[50]	E14 Retina	5 retinas	IgG	pAG-MN	C&R in PCR s	0.13	1559614
16	PRJNA678949	SRR13073031	[52]	LSK		IgG			10.11	5257074
17	PRJNA527826	SRR8745654	[54]	EILP	500	IgG	pA-MN	C&R with fixa	32.72	2023390
18	PRJNA682340	SRR13190129	[56]	E12.5 LGE	1000000	IgG	pA-MN	C&R with sec	7.76	31119152
19	PRJNA864644	SRR20727958	[58]	MEF	100000	"Input"	pAG-MN	C&R	4.24	282542
20	PRJNA493794	SRR7939979	[60]	MLL-AF9 leukemic cel	250000	IgG	pA-MN	C&R	6.19	2278008

Table S2: Fragments within Peaks (FRIP) scores for C&R blacklist regions within negative control samples after mapping with bowtie2 or bowtie

Human Number	FRIP bowtie2	FRIP bowtie	Mouse Number	FRIP bowtie2	FRIP bowtie
**1**	3.75	0.22	**1**	6.03	0.32
**2**	11.6	3.14	**2**	6.90	0.49
**3**	4.63	1.16	**3**	8.23	0.033
**4**	18.6	0.049	**4**	17.1	2.25
**5**	4.31	0.19	**5**	10.5	0.67
**6**	3.80	0.23	**6**	10.8	0.086
**7**	5.82	1.89	**7**	6.15	0.22
**8**	5.16	1.17	**8**	9.02	1.93
**9**	5.89	0.41	**9**	14.4	0.019
**10**	2.88	0.07	**10**	8.24	0.19
**11**	3.29	0.43	**11**	6.13	0.25
**12**	4.04	0.13	**12**	5.39	0.11
**13**	11.7	0.92	**13**	4.80	0.31
**14**	3.28	0.032	**14**	4.50	0.098
**15**	3.65	0.41	**15**	0.308	0.18
**16**	10.8	3.19	**16**	7.26	0.29
**17**	3.31	0.11	**17**	8.02	0.03
**18**	6.00	1.44	**18**	4.43	0.28
**19**	6.59	0.21	**19**	11.8	0.31
**20**	3.14	0.60	**20**	5.10	0.34
