# Supplementary material for: The CUT&RUN suspect list of problematic regions of the genome
Source: Genome Biol. 2023 Aug 10;24:185. doi: 10.1186/s13059-023-03027-3 (PMC10416431; doi:10.1186/s13059-023-03027-3)
Supplement: Supplementary file 3 — Additional file 3. Review history. [file 13059_2023_3027_MOESM3_ESM.docx]

**1^st^ Round**

**Reviewer 1**

Summary: Nordin et al. aggregate publicly available CUT&RUN "control" datasets (i.e. those intended to represent experimental background) to generate a "blacklist" in the style of the ENCODE Blacklist for ChIP-seq experiments. Using 20 datasets each from hg38 and mm10, they find that signal in blacklist regions is highly correlated across datasets, and that subtraction of blacklist regions from both publicly available and newly generated control datasets decreases their concordance. They go on to show that blacklist subtraction from experimental datasets representing diverse targets and provenance improves expected intra-target dataset correlations, indicating that the CUT&RUN blacklist is generalizable across experiments from different sources. Finally, they show that blacklist filtering prior to SEACR CUT&RUN peak calling improves apparent signal-to-noise and motif detection in experiments targeting Beta-catenin, Myc, and Max.

Evaluation: The authors support most of their conclusions in broad strokes. However, the manuscript could be improved with more quantitative evaluations of the improvement that results from blacklist usage (e.g. how much "closer" replicates become), including a clarification of the signal-to-noise analyses in Figure 4B-C. These points are important to confirm the utility of the blacklist for other users. There are also opportunities to glean more information about proper control experimental design (since the authors stress that this remains important), and to clarify some points on the sources of the background (which the authors speculate on at one point).

Impact: Just as the ENCODE blacklist has become a staple of ChIP-seq analysis, a high-quality CUT&RUN blacklist could be expected to have high utility among a growing user base.

Major Points:

- Figure 1B: For hg38, it appears that there is a "core" set of 15 control datasets that are most highly correlated across blacklist regions (> 0.8) with the remaining 5 (4, 20, 7, 10,13) showing lower correlation. Is there a pattern in correlation between the different "style" of control (e.g. IgG vs. HA vs. no antibody)? In the context of this comprehensive analysis, there's a nice opportunity to remark on which style most reliably reports blacklist regions so that users could have better guidance on what control to use for their experiments. Relatedly, a single mm10 dataset (15) seems to be very lowly correlated with the others—is there something distinct about this that would explain that?

- "We noticed that several false peaks are shared between the ENCODE and C&R blacklists (Figure 2B). We interpret that these artifacts are caused by genome assembly and mapping biases rather than the undesired purification and identification of unspecific DNA fragments". I don't quite follow here—isn't it possible that the same residual DNA could be carried over in both protocols? For instance, repeat sequences will by definition be more represented in background DNA, and there are mechanisms for it to be carried forward in both protocols (nonspecific antibody/bead binding in ChIP and spurious sequencing adapter ligation in CUT&RUN). Can the authors provide some evidence that these sites are indeed the result of assembly/mapping biases rather than physical carryover of background DNA? Unfortunately repeat sequences could contribute to both of these, so wouldn't rule either out.

- The authors might consider calculating statistics for intra- vs. inter-target distance (using all principal components) before and after filtering for Figure 4A in order to have a quantitative measure of the improvement. As it stands there may appear to be qualitative improvement, but it's difficult to judge.

- The average plot in Figure 4B is a little confusing because although the stringent-filtered peaks have more signal than the stringent unfiltered peaks (for instance), there is also higher signal outside the peak, indicating that the normalization may be inflating the total signal in some way. Can the authors comment on this, and whether this impacts the conclusions in Figure 4C (it's harder to see there, but the Max filtered dataset has higher non-peak signal than the Max unfiltered as well)? In any case, the increase in on-target motif content is more convincing for the argument presented here.

Minor Points:

- In Figure 1B, Speamans' Rho (I assume this is what's being indicated by the color) should be labeled next to the color bar.

- Although this is partly addressed in Figure 3, the authors should state clearly how many different cell types are represented in the control datasets they used, since this is relevant to the universality of the generated blacklists.

**Reviewer 2**

In this manuscript, Nordin et al present a CUT&RUN specific blacklist for hg38 and mm10 genomes, specifically for SEACR-based peak calling. Blacklists are widely used in genomic technologies, such as those provided by ENCODE for ChIPseq datasets. Therefore, this will be very useful to the community. I have the following comments/suggestions to improve the utility of these blacklists:

1. The authors perform the analysis using SEACR, but there are many other peak calling algorithms used, such as MACS2 and HOMER. On line 28-29, the authors state that they tested their blacklist filtering using MACS2, but found a lesser impact. However, no data is presented to this end, and the application on blacklists, or what is necessary with different peak calling algorithms would be extremely important for users.
2. The 40 datasets used for generating blacklists (and presented in Table 1) should include cell type and cell number.
3. The authors perform traditional CUT&RUN and their new modification, CUT&RUN-LoV-U. Is there any difference in blacklist locations using these two different techniques?
4. The authors discuss CUT&RUN using proteinA-MNase, although the majority of CUT&RUNs performed currently, to my knowledge, more often utilize proteinA/G-MNase. Is there any difference in data coming using these different tethering enzymes? Assessment of this is likely important for application.
5. The authors state that it as difficult to identify datasets that had negative controls, but I am surprised to hear only ENA was explored rather than also GEO. In a quick search, I found many more CUT&RUN datasets on GEO that could be easily identified for IgG or no antibody controls, and could be incorporated to strengthen the blacklists presented here.
6. While hg38 and mm10 are the currently most widely used genomes, mm39 has been more recently published and will become the more widely used mouse genome and T2T will become more widely used for human genome mapping. Therefore, to promote the most utility, it would be most beneficial to have blacklists generated for these two additional genome assemblies.

**Reviewer 3**

- The definition of black list regions is not very clear and seems to be peak-calling specific. It seems that it is just a set of artifactural peaks generated by SEACR during the CUT&RUN peak calling steps, at least this is what it seems from reading the paper, since pretty much all analyses including the discovery of black list regions come from the applying SEACR algorithm. If this is the case, the appearance of black list regions maybe itself due to SEACR algorithm, the insufficiencies of it, rather than a experimental targeting bias of the protocol.
The authors should demonstrate the existence of black-list regions from multiple peak callers, most notably MACS2. In my mind, MACS2 does not have many of the false-positivity issues associated with SEACR.
The black-list regions generated by this study should be broad enough to be applied to MACS2 peaks as well, not just SEACR.

- As a user, it is not clear what advantages the blacklist regions serve as compared to using individual study's IgG. If I have performed a CUT&RUN experiment along with a IgG experiment, I would expect that the blacklist peaks to be already included in the IgG and would be filtered away by the peak caller. Then what benefits would your blacklist regions serve? Here my study's IgG is also specific to my conditions, while blacklist regions are not.

- The definition of blacklist regions should be dependent on the mappability of the genome. Some regions of the genome, such as repetitive regions, are particularly prone to false positive peaks due to mappability and low quality genome assembly. We see a cluster of peaks in lowly mappable regions in mm10, hg19, etc. In my mind, this causes even greater issue and more widespread than the blacklist regions discovered in this paper.
Can authors consider including lowly mappable regions into their blacklist regions?
Authors most likely have missed these lowly mappable regions, as peaks in these regions may not be obvious in IgG but are quite obvious in real experiments. Hence, artifactual peaks that are not in IgG but in real experiments are not currently discovered.

- There are no explanations to how these blacklist regions might arise. The authors formulated a few hypotheses but none of them are tested in the paper. One obvious thing is that there is probably a sequence basis to blacklist regions, which should be characterized in this paper. Without this analysis, it would be hard to generalize blacklist regions to other genome assemblies, or model organisms.

- In building the compendium of CUT&RUN data for analysis, the selection of IgG limits the potential to discover artifactual peaks that are in real experiments but not in IgG.

- Ideally, we do not expect blacklist filtering to remove real biological signals. I wonder how many blacklist regions overlap with transcription factor binding sites, open chromatin regions, and DNA hypersensitive sites. Can authors perform de-novo motif analysis on these blacklist regions?

In summary, the paper may improve from clarifying the definition of blacklist regions, additional analyses into possible mechanisms by which blacklist regions arise, so that users may know how to deal with them computationally and experimentally.

**Authors’ response**

Detailed Response to Reviewers:

We are thankful for the referee’s thorough evaluation of our manuscript and data. Their criticisms and insights have helped us improve the strength of our manuscript and increase the utility of our blacklists for currently used and upcoming genome assemblies. Below please find a point-by-point response to each of the comments raised: each referee’s point (in black) is followed by our Response (in blue). Moreover, any changes made to the manuscript are highlighted in red and additions/changes to the figures are carefully reported.

Response to Reviewer #1:

Summary: Nordin et al. aggregate publicly available CUT&RUN "control" datasets (i.e. those intended to represent experimental background) to generate a "blacklist" in the style of the ENCODE Blacklist for ChIP-seq experiments. Using 20 datasets each from hg38 and mm10, they find that signal in blacklist regions is highly correlated across datasets, and that subtraction of blacklist regions from both publicly available and newly generated control datasets decreases their concordance. They go on to show that blacklist subtraction from experimental datasets representing diverse targets and provenance improves expected intra-target dataset correlations, indicating that the CUT&RUN blacklist is generalizable across experiments from different sources. Finally, they show that blacklist filtering prior to SEACR CUT&RUN peak calling improves apparent signal-to-noise and motif detection in experiments targeting Beta-catenin, Myc, and Max.

Evaluation: The authors support most of their conclusions in broad strokes. However, the manuscript could be improved with more quantitative evaluations of the improvement that results from blacklist usage (e.g. how much "closer" replicates become), including a clarification of the signal-to-noise analyses in Figure 4B-C. These points are important to confirm the utility of the blacklist for other users.

There are also opportunities to glean more information about proper control experimental design (since the authors stress that this remains important), and to clarify some points on the sources of the background (which the authors speculate on at one point).

Impact: Just as the ENCODE blacklist has become a staple of ChIP-seq analysis, a high-quality CUT&RUN blacklist could be expected to have high utility among a growing user base.

Response:

We thank the reviewer for their supportive take of our study, and for the careful attention to the analytical details in evaluating our manuscript. We have strived to improve the article with more quantitative details, as described below, and have taken the opportunity to examine our data more carefully in search of insights into control design and the origin of the blacklist regions.

Major Point 1:

Figure 1B: For hg38, it appears that there is a "core" set of 15 control datasets that are most highly correlated across blacklist regions (> 0.8) with the remaining 5 (4, 20, 7, 10,13) showing lower correlation. Is there a pattern in correlation between the different "style" of control (e.g. IgG vs. HA vs. no antibody)? In the context of this comprehensive analysis, there's a nice opportunity to remark on which style most reliably reports blacklist regions so that users could have better guidance on what control to use for their experiments. Relatedly, a single mm10 dataset (15) seems to be very lowly correlated with the others—is there something distinct about this that would explain that?

Response:

The point mentioned here is important and likely one that many readers of our manuscript would also find curious, so we are thankful for the chance to clarify it. We have now added a supplementary table (Supplementary Table 1), containing additional information about the datasets used, including type of control and other metrics such as protein used (pA-MN versus pA/G-MN), cell type and cell number, duplication and mapping rates. We have also created new supplementary figures (Supplementary Figure 1B and 1C) where we have marked the samples in the PCA plots based on different relevant categories (e.g., type of control antibody; CUT&RUN protocol), both before and after blacklist filtering, such that one could infer if any obvious clustering or trend exist based on each category. From this information we can deduce that the datasets that do not correlate as strongly with others across the human samples (namely 4, 20 7, 10 and 13) seem to have either high duplication rates and/or low number of mapped reads, possibly indicating lower library complexity. The exception is dataset 10 which seems to be, by all metrics considered, “normal”. Our conclusion is that the low complexity cannot easily be attributed to commonalities in any of the other parameters considered (the columns in Supplementary Table 1). Moreover, other equally sparse or duplicated datasets correlate much better with the others. There are likely other differences in how the experiments were performed or sequenced which cannot be easily pinpointed with such broad characterizations. A different conclusion can be drawn about the mouse dataset (15). Upon closer inspection, we noticed that this dataset is considerably different from the rest, and the vast majority of its reads map only to chromosome 1. While it also displays signal in the blacklist regions found in chromosome 1, there is markedly less on all the other chromosomes, a fact that, on its own, easily explains its low correlation with the other samples.

It is interesting to notice that, while most of the human datasets come from negative controls that made use of IgG, there is one exception in which an anti-GFP was used (number 5). Nevertheless, this sample is highly correlated with the others and not separated on the PCA plots. Also concerning the mouse data, there are mostly IgGs, one anti-HA, one “no-antibody” control and one labelled “input”; all these correlated well. In one of the original CUT&RUN Nature Protocols articles (10.1038/nprot.2018.015), it is stated that using a no-antibody control leads to a higher background:

as a result, it seems this is a rarely used option. To produce our internal blacklist using datasets from human HEK293T cells, we deliberately used equal numbers of anti-HA or IgG. While the PCA plot in Supplementary Figure 1C shows a slight trend of separation between the IgG and anti-HA samples before filtering, possibly suggesting a bias caused by the negative control used, this mostly disappears after filtering, showing that our CUT&RUN blacklist accounts for an entire spectrum of false positive peaks that appear both when IgG or anti-HA are used.

We wish to add that, in our experience, using for example an anti-HA vs IgG may lead to a lower CUT&RUN DNA yield, but when these samples are sequenced to the same depth the artifacts and coverage are similar. We have added an explanation about the mouse dataset number 15 in the figure legend, and a discussion section about the different factors that could affect the outcome of control samples to the revised manuscript.

Major Point 2:

"We noticed that several false peaks are shared between the ENCODE and C&R blacklists (Figure 2B). We interpret that these artifacts are caused by genome assembly and mapping biases rather than the undesired purification and identification of unspecific DNA fragments". I don't quite follow here—isn't it possible that the same residual DNA could be carried over in both protocols? For instance, repeat sequences will by definition be more represented in background DNA, and there are mechanisms for it to be carried forward in both protocols (nonspecific antibody/bead binding in ChIP and spurious sequencing adapter ligation in CUT&RUN). Can the authors provide some evidence that these sites are indeed the result of assembly/mapping biases rather than physical carryover of background DNA? Unfortunately repeat sequences could contribute to both of these, so wouldn't rule either out.

Response:

We appreciate the chance to clarify the text and correct our oversight. It is entirely correct that it is possible that similar residual DNA could be carried over in both protocols – especially repeat sequences which are common in the genome. We have changed the text to reflect this. To determine the source of these types of artifacts would be difficult, though the fact that subsequent genome releases contain fewer artifactual signal sites (doi.org/10.1038/s41598-019-45839-z) implies that the pile-up of repeat sequences in narrow regions, causing artifacts, are at least partially computational in nature. Some clue can be gleaned from our new blacklist compiled from the same data but applicable to the most recent telomere-to-telomere (T2T) human genome. Our resulting T2T genome blacklist, for instance, contains only 212 blacklist regions versus the 1049 for the hg38 blacklist, indicating that severalregions (repetitive and/or with lower mappability) that caused computational issues were resolved in the new assembly, resulting in a greatly reduced artifactual signal buildup.

Major Point 3:

The authors might consider calculating statistics for intra- vs. inter-target distance (using all principal components) before and after filtering for Figure 4A in order to have a quantitative measure of the improvement. As it stands there may appear to be qualitative improvement, but it's difficult to judge.

Response:

We thank the reviewer for the suggestion of a quantitative way to validate our observations. To be consistent with the visualization in the PCA and to quantify more precisely the analytical outcome shown in the figure, we calculated the pair-wise Euclidian distances for the samples in Figure 4A before and after filtering using principal components 1 and 2, which are sufficient to explain the almost entire variation before filtering, and the largest fraction of the variation after filtering. We defined three groups for each experiment, depending if targeting i) β-catenin; ii) TCF/LEF, and iii) histone modifications (H3K27ac for the hindlimb mouse data, H3K4me and H3K4me2 for HEK293T human data), since these factors are expected to yield similar DNA-binding patterns(note: while we are aware that β-catenin binds the genome via TCF/LEF, the b-catenin profiles are typically less clean, and should be grouped separately). We then considered the average intra- and inter- target distances for each group, before and after filtering. In the human HEK293T dataset, before filtering, the average intra- target distance was 0.128 and inter-target 0.343. After filtering, these values shifted to, respectively, 0.091 (a decrease, indicative of experimental precision – higher intra-group concordance) and 0.431 (an increase, suggestive of specificity – higher inter-group heterogeneity likely due to differential target’s biological activity). In the mouse hindlimb, before filtering, the intra-target average was 0.159, and inter- was 0.462, which when filtered became 0.061 and 0.517, similarly to the human example. In both cases, the intra-sample distances decreased, while the inter-sample distances increased. The ratio between inter- and intra- target distance increased from 2.68:1 to 4.73:1 in HEK293T, and from 2.18:1 to 8.53:1 in the hindlimb, numbers that allow us to provide a careful quantitation of the resulting increase in the sample heterogeneity.

Based on this suggestion and to make the manuscript as a whole more detailed, we also have added Scree plots for all PCAs in the figures, as a more quantitative description of how the Eigenvalues of the calculated principal components that contribute to the cumulative variability change. From this, it becomes clear that removing the regions included in the blacklists dramatically lowers the Eigenvalue of PC1, thereby increasing the contribution of the remaining principal components on the total variance observed – a clear measure of the augmented detection of inter-sample biological variability.

Major Point 4:

The average plot in Figure 4B is a little confusing because although the stringent-filtered peaks have more signal than the stringent unfiltered peaks (for instance), there is also higher signal outside the peak, indicating that the normalization may be inflating the total signal in some way. Can the authors comment on this, and whether this impacts the conclusions in Figure 4C (it's harder to see there, but the Max filtered dataset has higher non-peak signal than the Max unfiltered as well)? In any case, the increase in on-target motif content is more convincing for the argument presented here.

Response:

This is an interesting observation which, we believe, could have arisen from multiple factors. First, it is important to note that the average plots in Figure 4B represent the signal intensity of a relatively small window of genomic space surrounding the peaks and measure the local background in the immediate proximity to the peak summits – that is, do not see the global background. In fact, the number of pair- end reads in the unfiltered bam file (containing all mapped reads) is 7,127,741, while that in the blacklist-filtered bam file it is 6,936,126. As the difference is small (<3% of the total reads), the background signal surrounding the peaks is likely not due to a general inflation of the total signal caused by blacklist filtering. The average width of the peaks for β-catenin increases with increasing stringency, and this rises with the level of background. Average peak width spans from 1117 bp (relaxed), to 1421 and 1461 (relaxed filtered and stringent, respectively), to filtered (1850). However, the average profile graph normalizes this, and the scale on the X-axis represents the % of peak width, as opposed to a set number of base pairs. This is an appropriate representation for SEACR, which is empirically driven and defines peak width based on existing breaksin coverage (the zeroes at the side of the curves). The profile is of course just an average of many peaks that range in size, breadth and topology, and may not fully represent the differences between the peak sets.

Prompted by this referee’s comment, we reasoned that it could make sense that higher stringency peaks become broader (larger area under the curve), and that wider peaks could indicate regions displaying an overall denser coverage. As the number of peaks gets drastically smaller, it is possible that a small set of high confidence peaks (likely called in all datasets) have much higher local signal, and this could affect the average signal more strongly in a smaller dataset than it would in a larger one.

To test this, we chose 4 large regions which consistently contain more signal in our CUT&RUN experiments in HEK293T; from the pattern of coverage on the genome, these are likely duplicated genomic traits in our cell population. Since SEACR relies on global background, it does not adjust for this type of increased regional signal (this would not be necessary, as eukaryotic cells are typically perfectly diploid) and as a result, many more peaks are called in these regions. This does not mean that they are not true peaks, and we believe that some constitute real biological signal. Only for the sake of this analytical test, we removed all the peaks occurring in these regions from all four peak sets, and re-ran the average signal profile. In this plot, which is now included in Supplementary Figure 1D, the background signal is much more concordant between peak sets, while the increased signal in the filtered peak sets is retained, or even slightly increased when filtered more stringently. This is likely due to the fact that the smaller peak sets were more highly biased by the high-signal regions: peaks in these regions accounted for 18% in relaxed, 35% in filtered relaxed, 38% in stringent, and 61% in filtered stringent. This makes it clear that the increased background was due to an increased prevalence of high signal regions, and not due to global signal inflation occurring after removing the blacklist regions. We have included our best explanation of this observation in the description of this analysis in the revised figure legend.

Since in 4B the difference in peak signal is larger than the difference between the background and, as pointed out, the increase in on-target motif prevalence backs the conclusions that the filtered peaks are more trustworthy, we believe that the conclusions are not strongly affected by the increase in background. And, as shown above, the normalization of the background when high-signal regions were removed from the peak sets enforces that the increased background was not due to the blacklist inflating total signal. Regarding 4C (now 4B, right panel), the background for MAX may also be slightly higher in the filtered datasets, but the difference in peak signals is much stronger than the possible confounding affects from the increased background. It is possible that K562 cells, used in the MYC and MAX experiments, which are known to be aneuploid with broad polyploid chromosomal traits (DOI: 10.1016/s0301-472x(00)00558-0), also have high-signal regions similarly to HEK293T and this could cause the apparent slight increase in background signal.

Minor Point 1:

In Figure 1B, Speamans' Rho (I assume this is what's being indicated by the color) should be labeled next to the color bar.

Response:

We have now added the label for Spearman’s Rho in Figure 1B.

Minor Point 2:

Although this is partly addressed in Figure 3, the authors should state clearly how many different cell types are represented in the control datasets they used, since this is relevant to the universality of the generated blacklists.

Response:

We have added this more clearly to the manuscript text and added the exact cell type information for all datasets used in Supplementary Table 1.

Response to Reviewer #2:

In this manuscript, Nordin et al present a CUT&RUN specific blacklist for hg38 and mm10 genomes, specifically for SEACR-based peak calling. Blacklists are widely used in genomic technologies, such as those provided by ENCODE for ChIPseq datasets. Therefore, this will be very useful to the community.

I have the following comments/suggestions to improve the utility of these blacklists:

Response:

We are grateful for the reviewer’s positive view of the potential impact of our manuscript for the community and have made changes to improve it based on the suggestions below.

Point 1:

The authors perform the analysis using SEACR, but there are many other peak calling algorithms used, such as MACS2 and HOMER. On line 28-29, the authors state that they tested their blacklist filtering using MACS2, but found a lesser impact. However, no data is presented to this end, and the application on blacklists, or what is necessary with different peak calling algorithms would be extremely important for users.

Response:

We agree that since other peak callers, notably MACS2, are often used for CUT&RUN, it is important to understand how the blacklists impact on them. We have now added similar analyses testing the blacklist filtering on the MACS2 peak calling as was done with SEACR, and show them in the revised Figure 4C. Specifically, we applied MACS2 peak calling and display the same β-catenin dataset both before and after filtering our blacklisted regions. Performing peak-calling on the unfiltered datasets against the individual experimental control resulted in blacklist regions being called as peaks (19 regions, representing 6% of called peaks), showing that even though MACS2 uses local background to identify regions of enriched signal, artifact peaks can still “slip through” even when meeting the stringent signal-to-local-noise ratio requirements of MACS2. Additionally, the blacklist-filtered dataset had slightly more peaks called at the same stringency once blacklist peaks were retrospectively removed (294 versus 292). When compared with average profiles, the signal normalized per million reads was higher in the filtered set, likely because of a reduced total signal after removal of all the readsfalling into blacklist regions. Next, we tested MACS2 for MYC and MAX, and saw the same general pattern of improvements: from blacklist regions being called in the unfiltered datasets (84 and 96 regions, respectively) and removed by our blacklist, to increased number of peaks and a stronger average profile in the filtered datasets. While these effects are not quantitatively as drastic as we find them to be with SEACR, we show that blacklist filtering of CUT&RUNs analyzed with MACS2 is advantageous, whether the filtering is performed before or after peak calling to remove spuriously called artifactual signal.

Point 2:

The 40 datasets used for generating blacklists (and presented in Table 1) should include cell type and cell number.

Response:

We have now added a supplementary table (Supplementary Table 1) containing additional information about the datasets used (when the information is available), including cell type, cell number, antibody used, MN-based fusion protein, protocol, PCR duplication rate, and total number of mapped reads. However, the cell number was not always evident from the data repository and methods section of the selected studies. In these cases we decided to leave the corresponding space blank.

Point 3:

The authors perform traditional CUT&RUN and their new modification, CUT&RUN-LoV-U. Is there any difference in blacklist locations using these two different techniques?

Response:

This is an interesting question brought up by the reviewer. For our internally generated blacklists, we used data from both traditional CUT&RUN and CUT&RUN LoV-U in roughly equal numbers. Information about which protocol was used is now included in Table 2. Overall, we have not seen evident differences between the two protocols in regard to artifactual signal generated by the negative control. We performed dedicated PCA analyses to quantify any potential contribution of the protocol employed and show thisin the revised Supplementary Figure 1C (where the samples are colored based on protocol and their distribution shown before and after filtering): there are not clear differences in where the samples are distributed on the plots.

Point 4:

The authors discuss CUT&RUN using proteinA-MNase, although the majority of CUT&RUNs performed currently, to my knowledge, more often utilize protein A/G-MNase. Is there any difference in data coming using these different tethering enzymes? Assessment of this is likely important for application.

Response:

It is true that we did discuss pA-MN in the text, though we typically use pA/G-MN, which is also likely more used currently, as suggested by the reviewer. In fact, the datasets used to build the blacklists were generated with datasets obtained by the use of both enzymes: 12 pA-MN and 8 pA/G-MN in human, 7 pA-MN and 12 pA/G-MN (1 unknown) in mouse. Additional PCA plots with samples colored based on the protein variant used can be now found in Supplementary Figure 1B, and from these one could conclude that no obvious differences in sample clustering are caused by the use of one or the other protein. We have also amended the text to reflect that both proteins can and are used for CUT&RUN.

Point 5:

The authors state that it as difficult to identify datasets that had negative controls, but I am surprised to hear only ENA was explored rather than also GEO. In a quick search, I found many more CUT&RUN datasets on GEO that could be easily identified for IgG or no antibody controls, and could be incorporated to strengthen the blacklists presented here.

Response:

The reviewer is entirely correct in that there are more datasets which could be included. However, as explained below, we do not consider this necessary, at this stage. Regarding searching the ENA, the contents of the ENA and GEO databases are at least partially redundant, as each contain the entirety of the SRA (Sequence Read Archive). For this study, we searched on the ENA platform. Of course, we cannot exclude that for other users the data files are more clearly labelled on GEO. While we did not search through all of the possible datasets, we strived to select negative control experiments clearly labelled that we considered representative of a broad spectrum of conditions. To this aim, for example, we overlooked many datasets that had similar characteristics to datasets that were already including in the blacklists. Moreover, we tried to ensure that no two datasets were from the same cell line or laboratory. The reason why we considered our search to be sufficient was based on the following observation. We originally built the blacklists on fewer datasets – yet these blacklists performed already surprisingly well on most of the CUT&RUN peaks sets tested. We then decided to add more dataset to compile the blacklists, similarly to how this referee is suggesting, until we reached the current number of 20. We could observe that, in doing so, we gradually recovered more blacklist regions, yet the increment was relatively small, strongly suggesting a saturation effect, and that the CUT&RUN blacklisted regions are consistently found across diverse control experiments. This led us to believe that the blacklists generated where “mature” and would not necessitate a further increase in datasets inclusion. Additionally, the very fact that our experimentally generated blacklists compiled from only 8 total (blacklist regions called in >=3 of 8) of our in-house, cell type specific datasets was able to recapitulate > 80% of the blacklist peaks (83% in human, 87% in mouse) reinforces our conclusions.

Point 6:

While hg38 and mm10 are the currently most widely used genomes, mm39 has been more recently published and will become the more widely used mouse genome and T2T will become more widely used for human genome mapping. Therefore, to promote the most utility, it would be most beneficial to have blacklists generated for these two additional genome assemblies.

Response:

We thank the reviewer for this suggestion; it will improve the utility of our paper in the future as we move to the newer genome assemblies. We have now generated blacklists using the same method and based on the same data but for the T2T and mm39 genomes and included this information in the manuscript (added them as supplementary files). Intriguingly, the T2T blacklist contains only 212 regions, in comparison to the hg38 1049 regions, likely because of the greatly improved assembly. The mm39 blacklist on the other hand, with 564 regions, is much more similar to mm10 with 559 regions, emphasizing the need to apply the CUT&RUN blacklist generated also on the updated reference genomes.

Response to Reviewer #3:

Summary: In summary, the paper may improve from clarifying the definition of blacklist regions, additional analyses into possible mechanisms by which blacklist regions arise, so that users may know how to deal with them computationally and experimentally.

Response:

We are grateful for the careful analysis of our paper by the reviewer, which has led us to performing additional analyses instrumental to better characterize (and understand) the blacklisted regions.

Point 1:

The definition of blacklist regions is not very clear and seems to be peak-calling specific. It seems that it is just a set of artifactual peaks generated by SEACR during the CUT&RUN peak calling steps, at least this is what it seems from reading the paper, since pretty much all analyses including the discovery of blacklist regions come from the applying SEACR algorithm. If this is the case, the appearance of black list regions maybe itself due to SEACR algorithm, the insufficiencies of it, rather than an experimental targeting bias of the protocol. The authors should demonstrate the existence of black-list regions from multiple peak callers, most notably MACS2. In my mind, MACS2 does not have many of the false- positivity issues associated with SEACR. The black-list regions generated by this study should be broad enough to be applied to MACS2 peaks as well, not just SEACR.

Response:

We originally built the blacklists using SEACR for the very reasons stated by the reviewer – SEACR does have drawbacks that render it more vulnerable to falsely identifying artifacts as true peaks, even if they are present in the negative control. Despite this, for many applications SEACR can be the optimal peak caller precisely due to the sparse nature of CUT&RUN data. Thus, we reasoned, the blacklists are particularly needed when SEACR is employed. By peak calling the negative controls themselves, and using this to build the blacklists, we could identify these regions and ensure that they can be properly removed while using SEACR. We also now clarify in the text how the blacklist was generated and why 30% (called peaks in >= 7 of 20 negative controls) was chosen as a threshold for false peak appearance in control samples (Supplementary Figure 1A). However, we agree that a useful blacklist would provide advantages regardless of the peak caller used. Therefore, we have now tested their use with MACS2 and added this analysis to the manuscript in the revised Figure 4C. We tested the effect of the blacklists on peak calling with MACS2 before and after filtering in a similar way as performed for SEACR. Calling the β-catenin unfiltered dataset against the control resulted in ca. 6% of called peaks falling into blacklist regions, showing that even though MACS2 uses local background, which often renders peak calling more stringent, artifact peaks do often “slip through”. The MACS2 peak calling improved upon application of our blacklists, as it conserved the same trues signal (the filtered dataset had 2 more peaks called at the same stringency, once the blacklist regions were retrospectively subtracted from the unfiltered peak set), and the global average signal profile, normalized per million reads, was higher in the filtered sets. The same pattern was observed for the MYC and MAX CUT&RUN experiments, where blacklist regions were called in the unfiltered datasets and filtered sets had higher average signal in the peaks, likely due to the decreased total background resulting from blacklist filtering.

As pointed out, MACS2 is often better at not identifying artifactual signal as peaks; however, we show that, despite this, blacklist filtering improves the performance of the peak algorithm in the datasets tested, and that filtering after peak calling invariably removes artifactual peaks that slipped past the stringent thresholding of MACS2. While the spurious peaks might not be many, and in very efficient experiments applying the blacklist might have little effect on the biological conclusions drawn, we believe that being more stringent and reducing the noise in a dataset as much as possible is always to be preferred.

Point 2:

As a user, it is not clear what advantages the blacklist regions serve as compared to using individual study's IgG. If I have performed a CUT&RUN experiment along with a IgG experiment, I would expect that the blacklist peaks to be already included in the IgG and would be filtered away by the peak caller. Then what benefits would your blacklist regions serve? Here my study's IgG is also specific to my conditions, while blacklist regions are not.

Response:

We understand the reviewer’s criticism and have tried to clarify the manuscript text to better explain the advantages of using our blacklists. We designed the blacklists primarily to be a complement to the IgG or negative control used in a study. In an ideal experimental setup, all artifactual peaks would be enriched in every negative control, and peak callers would recognize these to filter them out. In our experience, however, this is not the case: very sparse negative controls may fail to identify artifacts that arise in the experimental tracks, and the peak callers may score these as enriched in the experiment vs. control and call them as peaks. Pre-packaged blacklists that identify common problematic regions that are present in CUT&RUN experiments with varying frequencies and filter them out will increase the confidence on the quality of the peaks found, as well as that in the downstream data analyses, such as motif recognition and gene ontology enrichment. To provide one fitting experimental example, we show below the analysis of some recent unpublished CUT&RUN from our lab, in which we performed 2 experimental replicates plus a negative control only, for we were limited by the scarcity of the tissue material employed. We identified an apparently convincing peak on the promoter of Gsk3b in both replicates (see IGV screenshot below). This peak was absent in the negative control. We subtracted the ENCODE mm10 blacklist and this peak remained. In conclusion, there was no reason to question this peak, and observing our candidate factor binding the promoter of such an important gene (GSK3 regulates the stability of a considerable fraction of the proteome) could have led to many further downstream experiments. After generating the CUT&RUN mm10 blacklist, we saw that this exact region was included in the blacklist – (de facto called in 8 out of 20 original datasets). In our case the anti-HA negative control did not identify this – for reasons on which we can speculate but that we do not fully understand. Overall, this served as an example for us, and exemplified the reason why we believe the CUT&RUN blacklists are useful tools to reduce false positives.

Additionally, as CUT&RUN can be in some cases performed on very little material, negative controls could be left out from experiments in models with scarce material to prioritize experimental samples. In these cases, we submit that the application of a blacklist would be essential.

Point 3:

The definition of blacklist regions should be dependent on the mappability of the genome. Some regions of the genome, such as repetitive regions, are particularly prone to false positive peaks due to mappability and low quality genome assembly. We see a cluster of peaks in lowly mappable regions in mm10, hg19, etc. In my mind, this causes even greater issue and more widespread than the blacklist regions discovered in this paper. Can authors consider including lowly mappable regions into their blacklist regions? Authors most likely have missed these lowly mappable regions, as peaks in these regions may not be obvious in IgG but are quite obvious in real experiments. Hence, artifactual peaks that are not in IgG but in real experiments are not currently discovered.

Response:

This is an important point, and a description of the reads mappability was overlooked in our original manuscript. In response we have characterized the blacklist regions based on their mappability using the UMAP tool (doi.org/10.1093/nar/gky677). Specifically, we took kmers of 36 bp and counted peaks that do not overlap with at least 1 uniquely mappable region, to find that 656/1049 (63%) in human and 42/559 (7.5%) in mouse are considered lowly mappable based on this definition. We did not include all of the lowly mappable regions in the genome in the blacklists, as doing this would lead to elimination of large sections of the genome; instead, we preferred to only focus on those regions which consistently become enriched in CUT&RUN negative control data. With subsequent genome assemblies, these mappability-derived artifacts are typically reduced. For example the ENCODE blacklist for hg38 contains less regions than hg19 when built on the same data due to the better assembly. Our new T2T C&R blacklist also shows this, having “only” 212 blacklist regions versus the 1049 present in hg38. In our opinion, a better way to reduce the problem of mappability bias in datasets where this poses a big issue, is to map reads to a masked genome with most of these marked as “NNNN”, or subtract lists containing all lowly mappable regions in the genome (which exist and are independent of the technique used). Of course however, this has the risk that real biological signal can be lost. In our experience, IgG/negative control tracks are typically as enriched in these low mappable regions as the experimental tracks. Admittedly, with very sparse IgG/negative controls (we find this more often occurring in CUT&TAG than in CUT&RUN) it can be difficult to get enough coverage and reads for these artifacts to arise, and thus they might be more present in the experimental samples but missed in the controls – hence called as peaks. This is one of the reasons that guided our selection of the experiments used in the assembly of our blacklists: the negative controls we chose here are in general high enough in coverage to detect many low-mappability artifacts, and this is evident by the prevalence of these regions in the blacklists generated.

Point 4:

There are no explanations to how these blacklist regions might arise. The authors formulated a few hypotheses but none of them are tested in the paper. One obvious thing is that there is probably a sequence basis to blacklist regions, which should be characterized in this paper. Without this analysis, it would be hard to generalize blacklist regions to other genome assemblies, or model organisms.

Response:

We have now characterized the blacklist regions and the underlying sequences to identify enriched sequences/motifs, genomic locations, etc., and this is described more thoroughly below in our response to your point 6. The characterization of the blacklist region annotations has also been added to Figure 1. While it would be difficult to identify the exact mechanisms for which artifacts arise, we have added a section to the discussion regarding this which can hopefully help to generalize our conclusions when other genome assemblies and model organisms are employed.

Point 5:

In building the compendium of CUT&RUN data for analysis, the selection of IgG limits the potential to discover artifactual peaks that are in real experiments but not in IgG.

Response:

We understand and appreciate the criticism from the reviewer, and we think this is an important point to consider. However, we have doubts concerning the real feasibility of these tests. In our opinion, building a blacklist on experimental data would have a much higher risk of considering biologically important regions, such as promoters or super-enhancers, as artifactual simply because they are enriched across different datasets. One should not, in other words, build a blacklist on the assumption that two different transcriptional regulators cannot occupy or co-occupy the same regulatory region. This is all the more relevant as CUT&RUN is most often performed on histone modifications, and thus the available data would likely be highly overlapping due to the inherent similarities of the experiments, caused by the known fact that, in a cell population, and often even in the same cell, a genomic position is decorated by a plethora of chromatin marks. To derive artifacts from experimental data would require the absolute certainty that two or many more factors do not ever bind the same loci, an assumption that would be – in our opinion – wrong to use. Moreover, we wish to point out that IgG is by far the most commonly used control, as we noticed from our database searches.

However, to avoid an IgG-driven bias in our blacklists, we have included controls other than IgG in the datasets, namely anti-HA, anti-GFP, and no-antibody samples. The anti-HA and anti-GFP are particularly useful, as in a way they model a knockout situation where a targeting antibody is used in a condition where its epitope is missing: this is often considered the gold-standard control of antibody (un)specificity across several assays, from immunohistochemistry to ChIP. We noticed that these controls, however, are not markedly different from the IgG samples, as seen in the PCA plots now shown in Supplementary Figure 1C, further indicating that all negative controls used yield (and contribute to) a comparable reduction of false signal.

Point 6:

Ideally, we do not expect blacklist filtering to remove real biological signals. I wonder how many blacklist regions overlap with transcription factor binding sites, open chromatin regions, and DNA hypersensitive sites. Can authors perform de-novo motif analysis on these blacklist regions?

Response:

It is a critical point made by the reviewer and we are grateful for the chance to address it. Adding more detailed information about the contents and sequence-information of the blacklists does improve the quality of our study. As a first step in assessing if the blacklists overlap with biological signal, we have annotated all these regions based on their genomic positioning (see graphs below). We found that both blacklists contained blacklist regions in promoters, 5’ or 3’ UTRs, TTS, and exon regions. While the mouse set is more enriched for most of these categories that typically can be associated to real signal, the human set was highly enriched for satellite elements and simple repeats, more easily recognizable as undesired results in a CUT&RUN list of peaks. Other regions identified by our blacklist are also enriched for intergenic loci, LINE, SINE and LTR annotations. We have added this characterization to the new Figure 1D and 1E and described them in the revised manuscript. hg38

Moreover, we have also performed known and de novo motif analysis using HOMER. In the human blacklist dataset, the only enriched known motif was CTCF. While this might appear an unexpected finding, it has been previously reported that repeated sequences and transposable elements are enriched for CTCF motifs (10.1016/j.cell.2013.02.029, 10.1101/gr.080663.108). In the mouse, there were no enriched known motifs. We consider these a significant validation of the regions identified by the blacklists, warranting their subtraction. Both blacklists resulted in a number of enriched de novo motifs, although the p-values were lower (more significant) and the motifs more prevalent in the human blacklist versus the mouse. Consistently, the human list is enriched for satellite regions, and these are known to contain transcription factor binding sites and promotor elements (REF 10.1159/000337116). For the referee’s revision, the top 10 de novo motifs for each blacklist are shown below.

hg38:

While the search of de novo motifs is bound to identify motif when given a DNA sequence as input – hence the tables above could themselves be an in silico artefact – the presence of motifs might also be indicative of genuine regulatory regions. There is always the possibility that, in some cases, blacklist filtering could remove real signal. For example, the aforementioned blacklist region in the promoter of Gsk3b in mm10 (our response to point 2) will certainly overlap with many real biological signals. However, we speculate that since the blacklist regions display a specific shape and are generally narrow, real biological enrichment on the Gsk3b promoter may be noticed as a shifted or broader signal, and differentiated from the artifactual peak region. Generally, however, we feel that it is best to err on the side of caution and risk false negatives at the expense of reducing false positives.

**2^nd^ round**

**Reviewer 1**

The authors have addressed my concerns satisfactorily.

**Reviewer 2**

The authors have addressed all my concerns and suggestions thoroughly as well as I believe the comments from the reviewers.

**Reviewer 3**

Thanks to the authors for performing analyses that were requested in my review.
Authors have successfully addressed some but not all of my concerns.

My remaining concerns are the following.

1) I am a bit bothered by the inconsistent style of presentation for the figures in the paper.
For example, in Figure 4 B and C, the presentation style is different for analysis made with SEACR than analysis made with MACS2.
In Fig 4B, the authors analyzed % of Peaks with LEF1 motif, TCF7 motif, TCF7L1 motif, and TCF7L2 motif for SEACR.
But in comparison with MACS2 (Fig 4C), the authors switched to P<0.001, peaks within blacklist, P<0.0001, so forth, and an entirely different dataset (MYC and MAX).

What is the reason for this inconsistency? Can't the authors measure motif presence, and peaks within blacklist for both SEACR and MACS2, and on a common dataset?
These inconsistencies prevent reviewers like me from getting an objective picture.

As a viewer, my immediate questions looking at these tables are:
- how many MACS2/SEACR peaks are there before filtering by blacklist?
- how many MACS2/SEACR peaks are there after filtering by blacklist?
- how has blacklist removal improve motif enrichment for each MACS2 and SEACR?

The current tables do not provide these basic information, but rather present some of the analyses in MACS2 and other analyses for SEACR.

2) The impact of the blacklist removal on user analysis is not clear.
There are only 1049 regions in human blacklist and ~500 for mouse blacklist.
For a typical CR experiment, more than 10,000 and 20,000 peaks are called - wouldn't removing 1000 regions have a relatively small impact on user's analysis, in the scheme of 10,000 peaks? This needs a better explanation in the main text.
If some of blacklist regions are quite large, then authors should mention that in the paper.
Judging from Fig 4B, it appears that removing blacklist reduced stringent SEACR b-catenin peaks from 2929 to 621, and MYC and MAX peaks from 7179 to 4229.

3) I am a bit concerned about the fact that blacklist regions are enriched in CTCF motifs, and several other motifs with high enrichment values (from authors de novo motif analysis). Eg. VDR, RELA, Hdx, PRDM1, PGR.
This implies that removing black list regions do come with the danger of removing true motif-enriched regions.
For a user who is actually doing a CTCF CUT&RUN, or VDR CUT&RUN (where the motifs tend to be enriched in blacklist), how much of impact does this removal make? Would it remove all CTCF peaks? What fraction of CTCF peaks are falsely removed?

This makes me worried that removal of blacklist regions may not be a recommendation for user doing specific CUT&RUN (like CTCF).
In order to reassure users, I feel that authors should evaluate on a broader set of CUT&RUN TF experiments (not just MYC/MAX or b-catenin examples already in paper) for impact on user's analysis, and provide key stats such as:
- peaks before filtering
- peaks remained after filtering
- fraction of peaks enriched for motif before filtering
- fraction of peaks enriched for motif after filtering

please do for SEACR and for MACS2.

This is the main remaining concern and I hope authors can spend a bit of effort in addressing this concern. Authors should select a diverse set of CUT&RUN TF experiments (>10 different TFs) to evaluate the impact.

4)
It would be great if authors can annotate each of blacklist regions for:
- whether it is low-mappability region
- characteristics: whether it is LINE, SINE, repeats, satellites, promoter, intergenic, etc
- which gene it closely maps to, if it maps to a promoter
- whether it also overlaps with the ENCODE blacklist region

This helps users easily understand the justification for the inclusion.

**Authors’ response**

We are thankful for the review of our manuscript and are pleased that reviewers 1 and 2 were satisfied with our revisions. We have now strived to address the remaining concerns of Reviewer 3, taking care to improve the presentation and consistency of the figures and to clarify the impact and possible risks of the blacklists in the revised text. We have now also provided a fully annotated version of the blacklists for readers to view and download with the paper.

Reviewer #1: The authors have addressed my concerns satisfactorily.

Reviewer #2: The authors have addressed all my concerns and suggestions thoroughly as well as I believe the comments from the reviewers.

Reviewer #3: Thanks to the authors for performing analyses that were requested in my review. Authors have successfully addressed some but not all of my concerns. My remaining concerns are the following.

Reviewer #3 Point 1:

I am a bit bothered by the inconsistent style of presentation for the figures in the paper. For example, in Figure 4 B and C, the presentation style is different for analysis made with SEACR than analysis made with MACS2. In Fig 4B, the authors analyzed % of Peaks with LEF1 motif, TCF7 motif, TCF7L1 motif, and TCF7L2 motif for SEACR. But in comparison with MACS2 (Fig 4C), the authors switched to P<0.001, peaks within blacklist, P<0.0001, so forth, and an entirely different dataset (MYC and MAX). What is the reason for this inconsistency? Can't the authors measure motif presence, and peaks within blacklist for both SEACR and MACS2, and on a common dataset?

These inconsistencies prevent reviewers like me from getting an objective picture.

As a viewer, my immediate questions looking at these tables are:

- how many MACS2/SEACR peaks are there before filtering by blacklist?

- how many MACS2/SEACR peaks are there after filtering by blacklist?

- how has blacklist removal improve motif enrichment for each MACS2 and SEACR?

The current tables do not provide these basic information, but rather present some of the analyses in MACS2 and other analyses for SEACR.

Response:

We thank the reviewer for pointing out this inconsistency which may make readers confused as to the affects of blacklist filtering on different peak callers. We have now strived to fix this oversight by presenting more complete, comparative tables that display motif presence and number of peaks on the same datasets to compare the outcomes of blacklist filtering when using either SEACR or MACS2. As the algorithms of SEACR and MACS2 are built differently, they are affected by filtering in slightly different ways, and so we originally tried in the first version to reflect the greatest effect of filtering on each peak caller in the tables. It is important to note that our intention was not to compare the two peak callers directly, nor draw any conclusion about which is more suitable for any given dataset.

In the revised version (Figure 4B, 4C) we now provide the full requested tables, including peaks before and after filtering regions within the blacklist, number of peaks within the blacklist in each set, and motif enrichment before/after filtering. This has been done for SEACR and MACS2, for two settings each, for all datasets analyzed. For space reasons, we have focused on only one motif per factor, and have reduced the beta-catenin comparison to just LEF1 (being the most abundant motif in the dataset). For MYC and MAX we chose the primary motifs as defined and identified by HOMER.

Reviewer #3 Point 2:

The impact of the blacklist removal on user analysis is not clear. There are only 1049 regions in human blacklist and ~500 for mouse blacklist. For a typical CR experiment, more than 10,000 and 20,000 peaks are called - wouldn't removing 1000 regions have a relatively small impact on user's analysis, in the scheme of 10,000 peaks? This needs a better explanation in the main text. If some of blacklist regions are quite large, then authors should mention that in the paper. Judging from Fig 4B, it appears that removing blacklist reduced stringent SEACR b-catenin peaks from 2929 to 621, and MYC and MAX peaks from 7179 to 4229.

Response:

We apologize for the apparent lack of clarity in the previous version and have edited the text to make the impact of blacklist filtering clearer. The reviewer is correct concerning the number of regions in the blacklists. Moreover, in both hg38 and mm10, the blacklist regions have an average size of approximately 5000 bp, so they are not exceedingly large. This information has been added to the text to make it more clear for readers. It is also true that some CUT&RUN experiments result in 10s of thousands of peaks (for abundant factors and histone marks). However, some more less abundant TFs and cofactors often only have thousands of peaks or even hundreds – in this case, removing 1000 could make a large difference. Especially when no negative control is used, large numbers of blacklist regions could be expected to be found among the peaks. Finally, while the precision of a dataset – the fraction of peaks that correspond to real biological events over the total signal (which might include blacklist regions) – typically improves for highly expressed factors or when good antibodies are available, we still consider of great importance removing as many false positives as possible. Even in a larger dataset, in fact, removing blacklist regions could make the difference between a motif or gene ontology term passing some statistical filtering or not, and thus affect whether it is considered significant.

These considerations refer to a workflow in which blacklist filtering is performed after peak calling. An alternative, well-accepted workflow implements blacklist filtering prior to peak calling. In this case, the enrichment in blacklist regions can affect the algorithm performance of the peak caller (mainly SEACR, to a lesser extent MACS2). We have now explained, in the revised manuscript, that: “These analyses indicate that blacklist filtering of bam files before peak calling has a larger impact on the final results when compared to filtering after peak calling with SEACR: the number of regions removed was much higher than the number of blacklist regions present in each unfiltered peak set (Figure 4B, center), confirming that the performance of SEACR is affected by the spurious signal in blacklist regions.”.

Figure 4B shows that filtering before peak calling (namely, removing blacklist region reads from the BAM files) has a greater impact on the peak calling than simply filtering out peaks that overlap blacklist regions, because it affects the normalization and/or the threshold that is empirically chosen by SEACR, and this occurs for both the relaxed and stringent settings. This is why the number of peaks changes from 2929 to 621 for beta-catenin, and from 7179 to 4229 for MAX – not by filtering thousands of peaks that overlap with the blacklist, but by removing the confounding affects of enriched blacklist regions on the SEACR algorithm itself.

MACS2, on the contrary, uses local background to threshold peaks, and therefore is much less affected. Despite this, we see changes, even if relatively minor ones, likely due to the fact that MACS2 normalizes the amount of total reads in the target dataset to the total reads in the control. We typically notice an increase in peaks called with the same stringency thresholds when filtering is performed prior to peak calling, and an increase in signal within the peaks (Figure 4C), while primary motif enrichment remains similar (the difference being less than 1%, either lower or higher after filtering). This indicates that filtering prior to peak calling can cause more peaks to be called (in total number) that contain the primary motif. The MAX dataset, with the highest FRIP score for the blacklist and consistently with SEACR, shows the largest difference after filtering.

We would like to point out that blacklist filtering is commonly employed for ChIP-seq (the 2019 ENCODE paper, doi: 10.1038/s41598-019-45839-z, has 420 citations) and even for CUT&RUN (for example the nf-core pipeline includes it). However, there seems to be no consensus in the field of whether filtering should be performed on the BAM files (prior to peak calling) or on the BED files after. To our knowledge, only one study (https://doi.org/10.3389/fgene.2014.00075) has compared the two choices, and concluded that blacklisted enrichment confounds MACS peak calling in ChIP-seq and thus filtering should be performed prior to peak calling. Our work adds a new layer of analysis that will help future experimenters to make their choice when interpreting CUT&RUN results.

Overall, with this study we aim to a) provide a CUT&RUN specific blacklist tailored to this technology, b) cover CUT&RUN specific regions that are missed in the ENCODE list while reducing the amount of the genome that is blacklisted as much as possible to avoid removing false negatives, and c) highlight the utility of filtering for blacklist regions before peak calling for CUT&RUN data, especially when SEACR is employed and results in overly high numbers of peaks.

Reviewer #3 Point 3:

I am a bit concerned about the fact that blacklist regions are enriched in CTCF motifs, and several other motifs with high enrichment values (from authors de novo motif analysis). Eg. VDR, RELA, Hdx, PRDM1, PGR. This implies that removing blacklist regions do come with the danger of removing true motif-enriched regions. For a user who is actually doing a CTCF CUT&RUN, or VDR CUT&RUN (where the motifs tend to be enriched in blacklist), how much of impact does this removal make? Would it remove all CTCF peaks? What fraction of CTCF peaks are falsely removed? This makes me worried that removal of blacklist regions may not be a recommendation for user doing specific CUT&RUN (like CTCF).

In order to reassure users, I feel that authors should evaluate on a broader set of CUT&RUN TF experiments (not just MYC/MAX or b-catenin examples already in paper) for impact on user's analysis, and provide key stats such as:

- peaks before filtering

- peaks remained after filtering

- fraction of peaks enriched for motif before filtering

- fraction of peaks enriched for motif after filtering please do for SEACR and for MACS2.

This is the main remaining concern and I hope authors can spend a bit of effort in addressing this concern. Authors should select a diverse set of CUT&RUN TF experiments (>10 different TFs) to evaluate the impact.

Response:

We would like to stress that this manuscript is not aimed to address the problems the field faces with peak callers, but rather to present CUT&RUN-optimized blacklist filtering as an option for users when potential detection of blacklisted regions could confound the peak calling or downstream analyses. Moreover, our blacklist – as any blacklist – might differently affect analytical pipelines of CUT&RUN performed on specific classes of transcription factors or on different tissues, and this issue would not be exhausted by testing our blacklist on tens of datasets. In this same spirit, the golden-standard ENCODE ChIP-seq blacklist was indeed presented in the 2019 paper with detailed testing on only one TF, REST.

Yet, having built our CUT&RUN blacklist on a wide range of negative controls, from different studies and backgrounds, and having tested its effects on peak calling on three different datasets, we are confident of its validity and wide applicability. Blacklists have been and are a fundamental, widely used tool for the analysis of ChIP-seq and CUT&RUN datasets and should be employed by users aware of their limitations and tailored to the actual analytical needs.

However, we have performed new analyses that we hope will alleviate the reviewer’s concerns about the finding of enriched motifs in our blacklist regions: Firstly, to address the specific concern about CTCF CUT&RUN, we have analyzed a CTCF sample and its negative control from the 2019 eLife CUT&RUN paper (https://doi.org/10.7554/eLife.46314). The CTCF dataset has a FRIP score of 2.5% after deduplication in the blacklist regions. Similar to the analyses shown in our manuscript on beta-catenin, MYC an MAX, blacklist filtering of BAM files reduced the number of SEACR called peaks and increased the percentage of motifs within the peaks on each setting. MACS2 peak calling also resulted in similar patterns as seen before, with similar or slightly higher numbers of peaks after filtering, and similar motif enrichment. Without filtering, blacklist peaks are called in all cases. All of the requested numbers are provided in the tables below. Our blacklists identify false positives in the dataset, but these represent a small fraction of the total peaks called. This shows that our blacklist does not remove the real CTCF signal.

SEACR:

RelaxedRelaxed FilteredStringentStringent Filtered

CTCFPrimary Motif77.29%86.07%82.29%90.58%

Number of Peaks1161825705982552

Blacklist Peaks430260

MACS2:

p001p001 filteredp0001p0001 filtered

CTCFPrimary Motif41.26%41.26%48.73%48.84%

Number of Peaks38148381842944229541

Blacklist Peaks13301170

Considering that CTCF typically binds ~50,000 regions per genome

(https://doi.org/10.1038/emm.2015.33), SEACR might not be the most ideal peak caller if one was attempting to identify all binding sites: even the relaxed unfiltered peak set may be far too stringent. In this case, while the filtering increases stringency (shown by increased motif percentage), this might cause loss of true peaks. But – and this is important – this would occur because of the change in SEACR determined threshold, not because there were thousands of peaks falling within the blacklist regions. These, in fact, were “only” 26 or 133, depending on peak caller, when called against the experimental IgG.

Paradoxically, we notice that sometimes the “cleaner” datasets from more abundant factors have less peaks called with SEACR, and vice versa for “dirtier” datasets for more rare factors. We had already stated in the text that if SEACR is performing well, it might not be needed to filter the blacklist prior to peak calling: in these cases, it can be performed downstream. We have now emphasized this more with a warning that when SEACR is already being highly stringent, it may not be advisable to filter the blacklist before peak calling as this can increase the stringency further. However, we have also seen the opposite: that when no peaks at all are being called (on a clearly working dataset), blacklist filtering can rescue this.

Secondly, curious to knowing whether the ENCODE blacklists also contained genuine motifs for TFs, we have performed the same known and de novo motif search on them. We found that the ENCODE blacklists present an even higher enrichment in various motifs than our blacklists, with many known and de novo hits for both hg38 and mm10 lists. The hg38 list had 24 significant known motifs (q < 0.05) including hits like FOX, PAX, GATA, and TCF factors, while the mm10 list had > 100 significant known motifs. For comparison, the only known hit for our blacklist was CTCF, and there were none for the mouse list. Despite their motif enrichment, the ENCODE blacklists are commonly used. We show below the top 10 enriched motifs for each ENCODE blacklist below:

*** SEE IMAGES IN THE ATTACHED WORD FILE

Motifs are very common in the genome, and as has been reported previously, there are many more occurrences of a motif in a genome than there are binding events of any given factor (as seen by ChIP-seq or CUT&RUN). Therefore, filtering out a motif- containing region does not necessarily imply losing any real biological signal. Additionally, as our blacklists were built on negative controls, we would like to emphasize that even if a peak is found in a TF dataset in one of these regions, it should not be trusted unless additional orthogonal evidence of regulation is provided.

Reviewer #3 Point 4:

It would be great if authors can annotate each of blacklist regions for:

- whether it is low-mappability region

- characteristics: whether it is LINE, SINE, repeats, satellites, promoter, intergenic, etc

- which gene it closely maps to, if it maps to a promoter

- whether it also overlaps with the ENCODE blacklist region

This helps users easily understand the justification for the inclusion.

Response:

We thank the reviewer for pointing out a useful complement to the information we provided. We now include a supplementary excel file with sheets for each blacklist containing the region coordinates, region annotation, gene annotation to closest TSS, mappability, and whether it overlaps with the ENCODE list.

**3^rd^ round**

**Reviewer 3**

The authors have performed important evaluations according to my suggestions.
However, upon careful look, I discovered major issues in how the authors are performing CUT&RUN analysis.
The main issue lies in the overall low number of peaks discovered by SEACR as analyzed by authors, which raise doubts about SEACR processing steps.

For example, authors have found only 552, 2570 peaks in the re-analysis of public CTCF CUT&RUN data. This is unusually low number of CTCF peaks and suggest flaws during SEACR peak calling steps. Even in the stringent case, there are only 5982 CTCF peaks discovered where the number of expected CTCF peaks should be in the 30,000-50,000 range.

Similarly when looking at MYC, MAX CUT&RUN in Figure 4B also revealed very low number of SEACR-called peaks (only 1600 MYC peaks and 1500 MAX peaks in the stringent case).

I therefore do not think that the authors are using SEACR correctly for peak calling. MACS2 statistics are more believable.

I suggest that the authors carefully review the CUT&RUN processing steps as outlined in the original CUT&RUN paper (Skene et al 2017), and get to the bottom of why the number of peaks are so low.

One possibility is that authors are choosing a very stringent significance cutoff which produced very small number of SEACR peaks. But I do not think this is the default behavior of SEACR.

In any case, the primary motif % in Fig4B, 4C is meaningless when there is only a very small number of peaks called by SEACR.
A fairer comparison is to present the primary motif % at the number of peaks equal to that of MACS2.
The current presentation of result (showing a high % of motif in SEACR, but a very low number of peaks) is very confusing and misleading (Fig4B, 4C). Such a low peak number is a sign that the CUT&RUN experiment does not work, and should not be used for analysis.

**Further comments from Reviewer 2**

I do think this paper will be helpful to the community, but the discrepancy between SEACR and MACS2 is a bit difficult to reconcile. I am happy that they included it in the paper as it is more helpful this way.

Yes, so SEACR has a far too stringent peak calling threshold. Many of us don't use it because of this stringency, which honestly loses pretty solid peaks that HOMER or MACS2 (or by eye) would be called.

So I don't think that it is a concern that the CTCF calls are so low; based on everything I have experienced and heard about SEACR, it is normal.

And the key here is that the authors are being transparent about everything, so it is totally fine from my perspective.

That being said, the blacklists seem to be necessarily specifically made for the peak calling algorithm used. And so the same lists shouldn't be used for SEACR vs MACS2.
